# Genome-wide associations identify novel candidate loci associated with genetic susceptibility to tuberculosis in wild boar

**DOI:** 10.1038/s41598-018-20158-x

**Published:** 2018-01-31

**Authors:** João Queirós, Paulo Célio Alves, Joaquín Vicente, Christian Gortázar, José de la Fuente

**Affiliations:** 1 Centro de Investigacão em Biodiversidade e Recursos Genéticos (CIBIO)/InBio Laboratório Associado, Universidade do Porto, Campus Agrário de Vairão, R. Monte-Crasto, 4485-661 Vairão, Portugal; 2 Departamento de Biologia, Faculdade de Ciências da Universidade do Porto (FCUP), Rua do Campo Alegre s/n, 4169-007 Porto, Portugal; 3 SaBio, Instituto de Investigación en Recursos Cinegéticos IREC (CSIC-UCLM-JCCM), Ronda de Toledo s/n, 13071 Ciudad Real, Spain; 4 Wildlife Biology Program, University of Montana, Missoula, MT 59812 USA; 5 Department of Veterinary Pathobiology, Center for Veterinary Health Sciences, Oklahoma State University, Stillwater, OK 74078 USA

## Abstract

Tuberculosis (TB) affects a wide range of host species worldwide. Understanding host-pathogen co-evolution remains a global challenge owing to complex interactions among host genetic factors, pathogen traits and environmental conditions. We used an endemic wild boar population that had undergone a huge increase in *Mycobacterium bovis* infection prevalence, from 45% in 2002/06 to 83% in 2009/12, to understand the effects of host genetics on host TB outcomes and disease dynamics. Host genomic variation was characterized using a high-density single nucleotide polymorphism (SNP) array, while host TB phenotype was assessed using both gross pathology and mycobacterial culture. Two complementary genome-wide association (GWAS) analyses were conducted: (i) infected-uninfected; and (ii) 2002/06–2009/12. The SNPs with the highest allelic frequency differences between time-periods and TB outcomes were identified and validated in a large dataset. In addition, we quantified the expression levels of some of their closest genes. These analyses highlighted various SNPs (i.e. rs81465339, rs81394585, rs81423166) and some of the closest genes (i.e. *LOC102164072, BDNF/NT-3*, *NTRK2, CDH8, IGSF21*) as candidates for host genetic susceptibility. In addition to TB-driven selection, our findings outline the putative role of demographic events in shaping genomic variation in natural populations and how population crashes and drift may impact host genetic susceptibility to TB over time.

## Introduction

Tuberculosis (TB), which is caused by members of the *Mycobacterium tuberculosis* complex (MTC), affects a broad range of host species worldwide^1–3^. After thousands of years of host-pathogen co-evolution, MTC has become one of the most successful human pathogens in history^4–6^ and continues to be a global health emergence of overwhelming proportions (i.e. three people in the world die of TB every minute)^7^. Despite the fact that one-third of the human population may harbor this mycobacteria in an asymptomatic state^8^, only 10% of infected individuals develop clinical TB^9^. This inter-individual variation in susceptibility to TB is still poorly understood, although host genetic factors^6,10,11^, pathogen traits^4,5,12^ and environmental conditions^13–15^ have been reported to be the main drivers of distinct TB outcomes.

The effects of host genetics on susceptibility to TB have been documented in humans^6,10,11,16^, domestic animals^17–19^ and wildlife^20–24^. However, until recently most of these studies investigated plausible candidate genes rather than screening the whole genomic variation. Recent advances in genome sequencing, and particularly in the development of high-density single nucleotide polymorphism (SNPs) arrays, have improved genome-wide screening and, therefore, our ability to detect disease-associated gene variants without a priori knowledge of candidate genes possibly linked to phenotypes^25^. This whole genome strategy, which relies on the concept of linkage disequilibrium between polymorphic markers and chromosome segments, has allowed some progress towards the identification of gene variants associated with susceptibility to TB in humans^26–29^ and in cattle^17–19^. However, genome-wide associations (GWAS) have not yet been conducted in any reservoirs of MTC infection in the wild, which might currently represent the best natural system of host-pathogen coevolution^30^. While natural populations experience the selective mechanisms of pathogens, humans and domestic animals undergo medical care and testing-culling schemes that at some point disrupt their natural co-evolution.

The Eurasian wild boar (*Sus scrofa*) is considered the main wildlife reservoir of MTC infection, namely the *Mycobacterium bovis*, in the Mediterranean habitats of Iberia^31^. In this region, infection prevalence reaches more than 60% in wild boar, representing the highest prevalence of TB reported worldwide to date^32^. The wild boar is an interesting model species to study mycobacterial infection because it is highly susceptible to TB and reproduces some of the clinical signs observed in humans^31,33^. Infection occurs mostly in the first months of life through oral-nasal routes and the most frequently affected tissue are the mandibular lymph nodes through the formation of granulomatous lesions^34,35^. This is possibly the main organ responsible for disease progression (i.e. the dissemination of infection throughout the organism). Furthermore, recent findings have demonstrated that TB is responsible for 30% (on average) of the total mortality rate in adult wild boar, thus evidencing some degree of TB-driven selection^36^. Hunting and starvation caused by extreme environmental conditions (i.e. hot dry seasons) also contribute to wild boar mortality in Mediterranean ecosystems^37^. The environmental stressors induced by hot dry seasons coupled with strong MTC infection pressure, resulting from both high population abundance/aggregation and the indirect contact with MTC through a contaminated environment (i.e., waterholes)^38^, makes these wild boar populations an exceptional model with which to understand host-pathogen interactions. Under these conditions, the genetic capability of individuals to resist or succumb to MTC infection would be evidenced^21^. Previous studies have shown that environmental features, such as the type of management (i.e. fenced vs. open populations) and low levels of rainfall^32,39^, pathogen traits^12^ and host genetic effects^22,24,31^, influence the wild boar’s TB outcomes. Wild boar genetic variability^22^, namely methylmalonyl CoA mutase (*MUT*), complement component 3 (*C3*) and other innate and adaptive immune response genes, have been linked to host genetic susceptibility to TB^24,40–42^. Although earlier findings have improved our knowledge of the role of host genetics, they rely on plausible candidate genes rather than on whole genome approaches.

In order to increase our understanding of the genetic basis of host susceptibility to TB, we conducted a whole genome approach on a Mediterranean wild boar population endemic to *M. bovis* infection and for which a rising trend of TB prevalence has been observed throughout the last 10 years. We used a high-density SNP array (61565 SNPs) to characterize the genomic variation of infected and uninfected individuals from both the lowest and highest periods of TB prevalence (45% in 2002/06 and 83% in 2009/12). Additionally, transcriptomic analyses were conducted to quantify the mRNA expression levels of some genes close to differential SNPs identified on GWAS. Understanding how genomic variation fluctuates in natural populations and how it impacts on disease susceptibility over time is fundamental as regards imposing wildlife management strategies that will minimize and prevent disease outbreaks in nature. In most cases, disease prevention represents the sole approach by which to ensure the health status of natural populations. In addition, unraveling the wild boar genetic mechanisms involved in susceptibility to TB may lead to a better understanding of TB pathogenesis and facilitate the development of new strategies for the prevention and treatment of TB in humans and animals.

## Results

### Population demographic history

**Figure 1:**
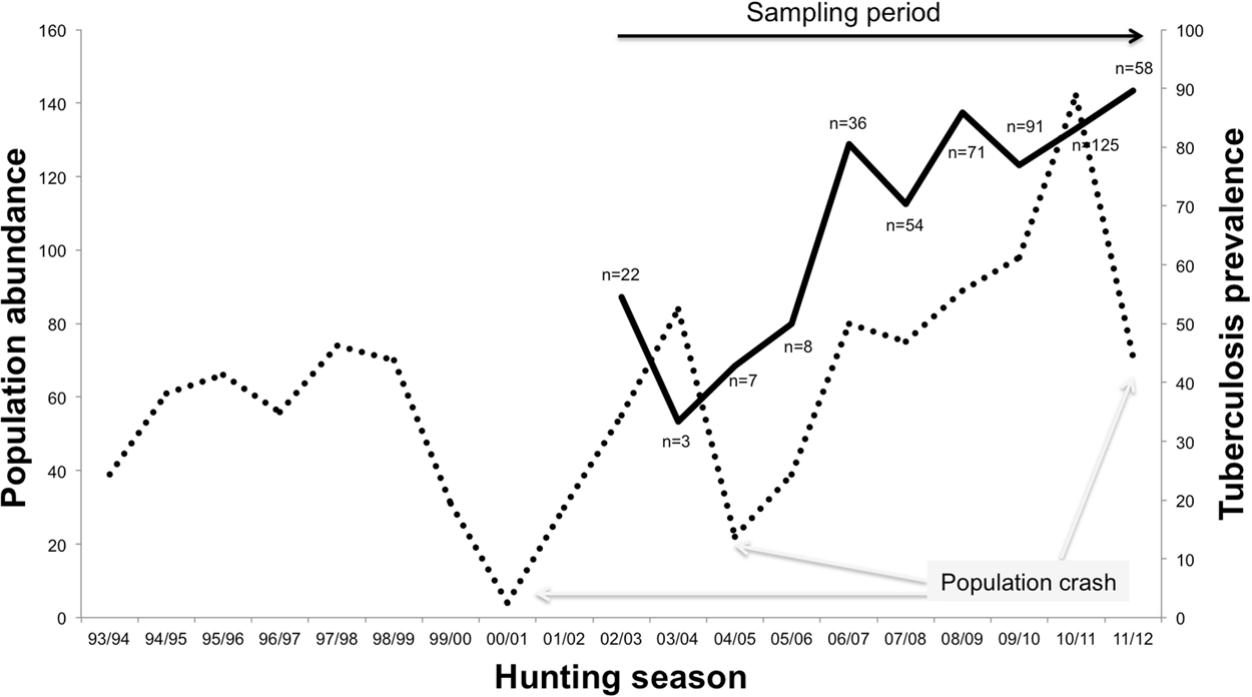
Plot showing the wild boar population abundance (dashed line) and tuberculosis prevalence (solid line) estimated for each season (number of individuals = #) throughout the monitored program implemented in the reserve and the sampling period, respectively. The three population crashes are also indicated.

**Figure 2:**
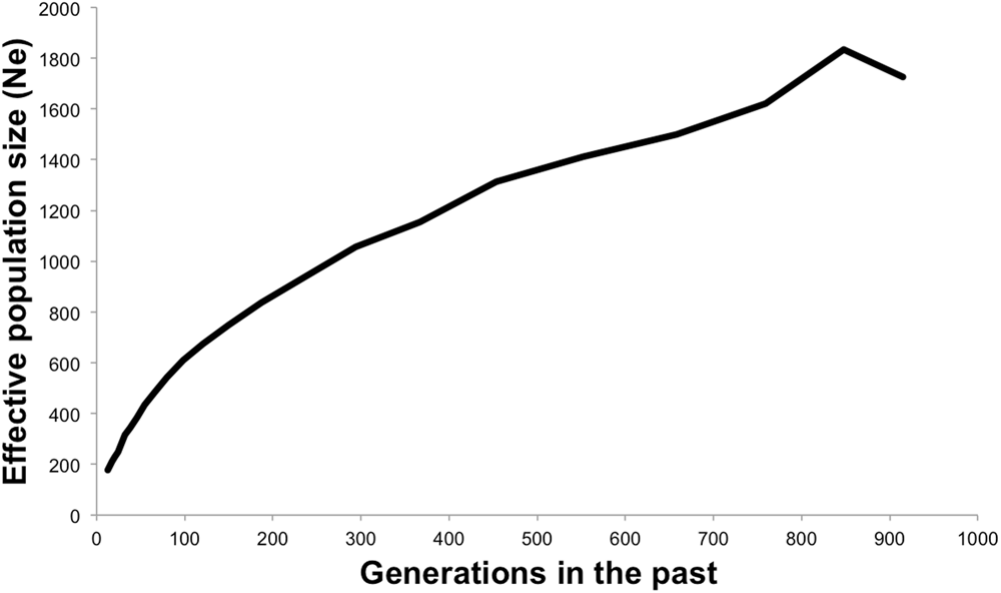
Historical trajectories of effective population size (N_e_) of the wild boar population inferred from genomic data for the past generations.

The number of individuals culled annually, which was used as a proxy of population abundance, showed that there had been three population crashes over the last 20 years (Fig. 1). A total of 1186 animals were culled, 755 of them (63.7%) in the last 10 years (Table S1). With regard to TB prevalence, an increasing trend was observed over time (Fig. 1). In the first four seasons (2002/03 to 2005/06), an average of 45% of the individuals analyzed had TB, which further increased to 83% in the last three seasons (2009/10 to 2011/12). The wild boar population studied represented a uniform genetic cluster and evidenced a lack of hybridization with commercial/domestic pig breeds and northern European wild boar populations in both PCA (Fig. S1) and STRUCTURE (Fig. S2) analyses. Furthermore, there was no evidence of population substructure within the sampled wild boar population, both when comparing infected/uninfected individuals (F_ST_ = 0.00) and individuals from different time-periods (F_ST_ = 0.00) (Fig. S3). Genome inflation factor calculations also revealed an absence of population substructure within our sampled population (Fig. S4). The historical perspective of effective population size (N_e_), calculated using the SNP data, showed a progressive decline in N_e_ in the past generations (Fig. 2).

### Genome-wide associations (GWAS), validation test and expression of candidate genes

**Figure 3:**
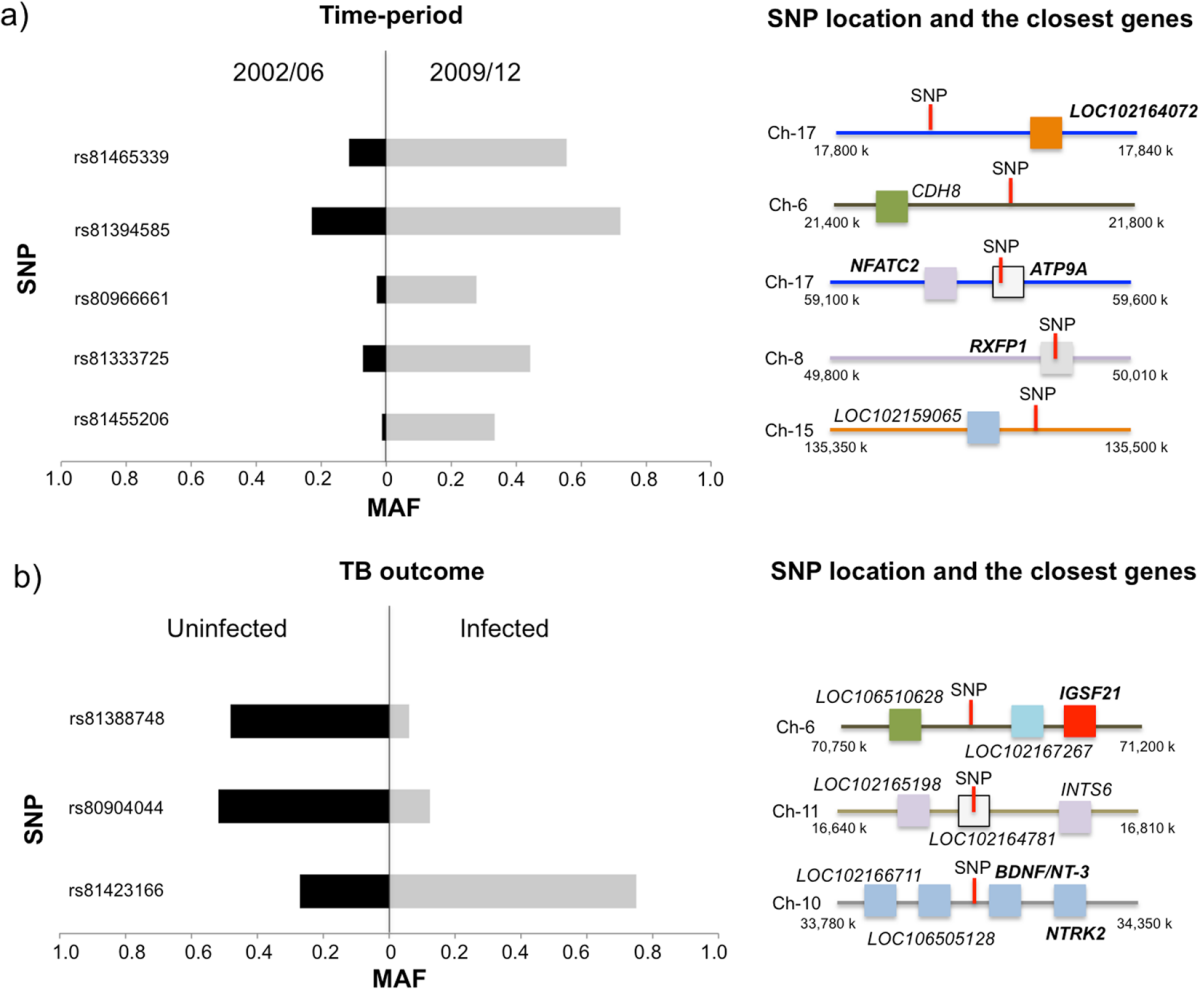
Minor allele frequencies (MAF) differentiation for the singular nucleotide polymorphism (SNP) identified in genome-wide association (GWAS) analyses. MAF differences are shown between (**a**) time-periods (2002/06 vs. 2009/12) and (**b**) tuberculosis (TB) outcome (uninfected vs. infected). The location of each SNP on porcine genome assembly *Sus scrofa* 10.2, and the closest genes are also represented. The candidate genes selected for mRNA gene expression analyses are indicated in bold type.

Genome-wide associations were conducted on individuals infected *vs*. uninfected with MTC and on individuals from the 2002/06 *vs*. 2009/12 time-periods. In each GWAS, we performed a standard case-control analysis and a stratified case-control analysis. In the latter analysis, we clustered the individuals by age class and time-period/TB outcome in order to account for their possible effects on statistical models. An empirical cut-off in the *p-values* distribution was assumed in GWAS (discovery stage) to select the highest differentiated SNPs, since none of the SNPs were significant after Bonferroni correction (*p-values* < 1.69E-06). The considered threshold (*p-value* < 1 × 10E-4), which represents the top 0.03% of the lowest *p-values* obtained, selected the eight highest differentiated SNPs for further validation (Fig. 3). In this analysis with a large dataset, some of these SNPs revealed statistically significant differences in allelic frequency between animal groups after Bonferroni correction (*p-value* = 6.25 × 10^−3^). In addition, the *p-values* of GWAS and validation tests were combined, and the initial conservative *p-value* of 1.69E-06 was considered as a threshold of significance. And finally, some of the genes close to the differentiated SNPs were further investigated in a large dataset using RNA expression. These findings are detailed described in the following sections for each GWAS.

#### Infected vs. uninfected individuals with MTC

**Figure 4:**
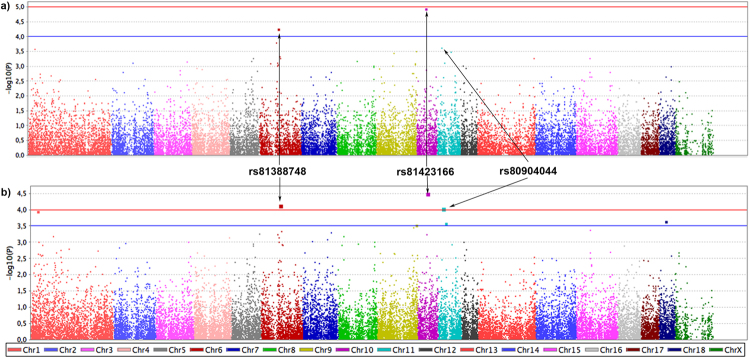
Manhattan plot displaying the genome-wide results [−log10(P)] of the standard (**a**) and stratified (**b**) association analyses between uninfected and infected individuals with *Mycobacterium tuberculosis complex (*MTC*)*.

The three SNPs (rs81423166, rs81388748 and rs80904044) with the highest divergent allelic frequencies (lower *p-values*) between MTC infected and uninfected individuals were initially selected from the classical GWAS (standard and stratified case-control analyses) (Fig. 4). When these SNPs were validated in a large dataset, the rs81388748 SNP was the unique that revealed a *p-value* below the considered threshold of significance (*p-value* < 6.25 × 10^−3^). By combining the *p-values* of GWAS and validation test, the rs81423166 SNP was the only one that showed a *p-value* < 1.69E-06. The polymorphism variant (A) of this SNP had lower odds of having TB (OR = 0.235–0.230, combined result for the standard and stratified analyses, respectively) (Table 1). This SNP, located on chromosome 10 of the porcine genome assembly 10.2, is flanked by various genes, including the BDNF/NT-3 growth factor receptor (*BDNF/NT-3*) and the neurotrophic tyrosine kinase receptor, type 2 (*NTRK2*) (Fig. 3). mRNA expression analyses of the these genes revealed statistically significant differences in gene expression between time-periods (Table 2), although no significant associations were found between SNP variants and gene expression (Table S2). Regarding to the rs81388748 SNP, and despite no significant result was found in the combined tests, the polymorphism variant (A) had high odds of having TB (OR = 5.116–5.189) (Table 1). Among the closest genes to this SNP, only one had a known biological function, the immunoglobulin superfamily member 21 (*IGSF21*) (Fig. 3). The expression of *IGSF21* gene was higher during 2002/06 (period of lower TB prevalence) than in 2009/12 (Table 2). In addition, the variant (A) had a significantly lower gene expression (mean = 0.270, 95% CI = 0.172–0.368) than the variant (C) (mean = 0.392, 95% CI = 0.305–0.479) (Table S2). Furthermore, a detailed expression analysis of the three previously described genes (*IGSF21*, BDNF/NT-3, NTRK-2) was performed considering the age class and time-period/TB outcome. This analyses revealed different gene expression patterns (Fig. S5 and Tables S3, S4 and S5), namely for *BDNF/NT3* significant differences were observed for juvenile/adult and infected/uninfected individuals between the time-periods (up-regulated in 2002/06).

**Table 1:** Results of the case-control analyses conducted to the eight SNPs that displayed the highest allelic frequency differences in the standard and/or stratified genome-wide associations (GWAS).

Case-control analyses	SNP	CHR	BP	A1	MAF	Test (*p-value* threshold)	Standard Analysis				Stratified Analysis			
							CHISQ	*p-value*	OR	CI_95%_	CHISQ	*p-value*	OR	CI_95%_
**2002/06 vs. 2009/12**	rs81465339	17	17811472	G	0.224	GWAS (1.00E-4)	17.14	3.48E-05	0.103	0.03–0.26	15.61	7.79E-05	0.082	0.02–0.32
						Validation (6.25E-03)	22.35	2.27E-06	0.144	0.06–0.34	23.87	1.03E-06	0.143	0.06–0.33
						Combined tests (1.69E-06)		9.30E-09	0.123			3.78E-09	0.128	
	rs81394585	6	21721506	A	0.329	GWAS (1.00E-4)	15.79	7.07E-05	0.114	0.04–0.37	15.03	1.06E-04	0.100	0.03–0.35
						Validation (6.25E-03)	16.96	3.82E-05	0.205	0.09–0.45	18.07	2.13E-05	0.202	0.09–0.44
						Combined tests (1.69E-06)		1.20E-07	0.168			6.66E-08	0.170	
	rs80966661	17	59400449	A	0.086	GWAS (1.00E-4)	12.14	4.92E-04	0.076	0.01–0.44	15.38	8.80E-05	0.028	0.00–0.29
						Validation (6.25E-03)	15.04	1.05E-04	0.112	0.03–0.40	13.50	2.38E-04	0.128	0.04–0.44
						Combined tests (1.69E-06)		9.89E-06	0.081			1.56E-05	0.107	
	rs81333725	8	49881116	C	0.158	GWAS (1.00E-4)	15.82	6.95E-05	0.096	0.03–0.35	12.14	4.94E-04	0.112	0.03–0.46
						Validation (6.25E-03)	6.38	1.16E-02	0.313	0.12–0.79	5.60	1.80E-02	0.346	0.14–0.85
						Combined tests (1.69E-06)		1.82E-04	0.229			9.49E-05	0.228	
	rs81455206	15	135428202	G	0.138	GWAS (1.00E-4)	19.91	8.14E-06	0.029	0.00–0.26	12.65	3.75E-04	0.022	0.00–0.33
						Validation (6.25E-03)	5.48	1.92E-02	0.340	0.13–0.87	5.70	1.69E-02	0.327	0.13–0.84
						Combined tests (1.69E-06)		2.44E-03	0.253			7.54E-04	0.224	
**Uninfected vs. Infected**	rs81388748	6	70996798	A	0.322	GWAS (1.00E-4)	16.23	5.61E-05	13.970	3.04–64.13	15.7	7.44E-05	13.860	2.90–66.28
						Validation (6.25E-03)	9.31	2.29E-03	4.454	1.62–8.93	9.68	1.86E-03	4.529	1.64–8.47
						Combined tests (1.69E-06)		1.95E-05	5.116			1.47E-05	5.189	
	rs80904044	11	16682857	G	0.408	GWAS (1.00E-4)	13.41	2.50E-04	7.519	2.33–24.26	15.3	9.18E-05	13.240	3.13–56.05
						Validation (6.25E-03)	0.63	4.29E-01	1.343	1.60–7.43	0.75	3.85E-01	1.393	1.65–7.22
						Combined tests (1.69E-06)		9.17E-06	4.639			7.09E-06	4.357	
	rs81423166	10	33956684	A	0.467	GWAS (1.00E-4)	19.18	1.19E-05	0.122	0.05–0.33	17.3	3.19E-05	0.119	0.04–0.34
						Validation (6.25E-03)	0.01	9.43E-01	0.280	0.16–0.65	0.01	9.07E-01	0.958	0.14–0.56
						Combined tests (1.69E-06)		1.56E-06	0.235			6.89E-07	0.230	

The Chi-square (CHISQ) value, odds ratios (OR) anzd 95% confidence interval (CI95%) of odds ratio are shown for the GWAS, validation test and combined tests.

Legend: CHR, chromosome code; BP, base pair position; A1, first allele code; MAF, minor allele frequency.

**Table 2:** Expression profile of genes associated to SNPs with the highest allele frequency differences in the standard and/or stratified genome-wide analyses (GWAS).

Gene	Time-period	N	Mean	SE	t-test	df	*p-value*	TB outcome	N	Mean	SE	t-test	df	*p-value*
**LOC102164072**	2002/06	23	0.63	0.046	5.2	53.9	0.000	Uninfected	21	0.48	0.057	1.1	49.1	0.268
	2009/12	35	0.29	0.046				Infected	37	0.39	0.053			
**RXFP1**	2002/06	26	0.89	0.081	3.4	56.8	0.001	Uninfected	22	0.74	0.094	1.0	48.4	0.324
	2009/12	35	0.50	0.078				Infected	39	0.62	0.080			
**ATP9A**	2002/06	4	0.00	0.000	−6.8	33.3	0.000	Uninfected	10	0.02	0.007	0.0	13.6	0.965
	2009/12	34	0.02	0.003				Infected	28	0.02	0.003			
**NFATC**	2002/06	7	0.08	0.020	0.1	8.8	0.904	Uninfected	14	0.07	0.014	−0.9	27.1	0.352
	2009/12	35	0.08	0.009				Infected	28	0.08	0.010			
**BDNF/NT3**	2002/06	26	0.86	0.068	8.9	35.3	0.000	Uninfected	22	0.49	0.069	0.1	56.6	0.899
	2009/12	35	0.20	0.031				Infected	39	0.48	0.076			
**NTRK2**	2002/06	24	0.02	0.003	4.0	43.3	0.000	Uninfected	21	0.02	0.003	0.0	46.3	0.990
	2009/12	33	0.01	0.002				Infected	36	0.02	0.003			
**IGSF21**	2002/06	26	0.57	0.044	6.7	52.7	0.000	Uninfected	22	0.39	0.052	0.9	53.2	0.351
	2009/12	35	0.18	0.037				Infected	39	0.32	0.050			
**MUT**	2002/06	26	1.11	0.065	2.7	58.4	0.009	Uninfected	22	0.98	0.078	0.2	48.8	0.854
	2009/12	35	0.86	0.069				Infected	39	0.96	0.067			
**C3**	2002/06	17	0.00	0.001	−5.6	38.5	0.000	Uninfected	17	0.01	0.003	−0.6	27.2	0.545
	2009/12	35	0.01	0.002				Infected	35	0.01	0.002			

Differences between individuals from different time-periods and tuberculosis (TB) outcome were determined using an independent t-test. Detailed results for age class and time-period/TB outcome are provided in the Supplementary Information.

Legend: N, number of individuals; SE, standard error of the mean; df, degrees of freedom.

#### 2002/06 vs. 2009/12 time-periods

**Figure 5:**
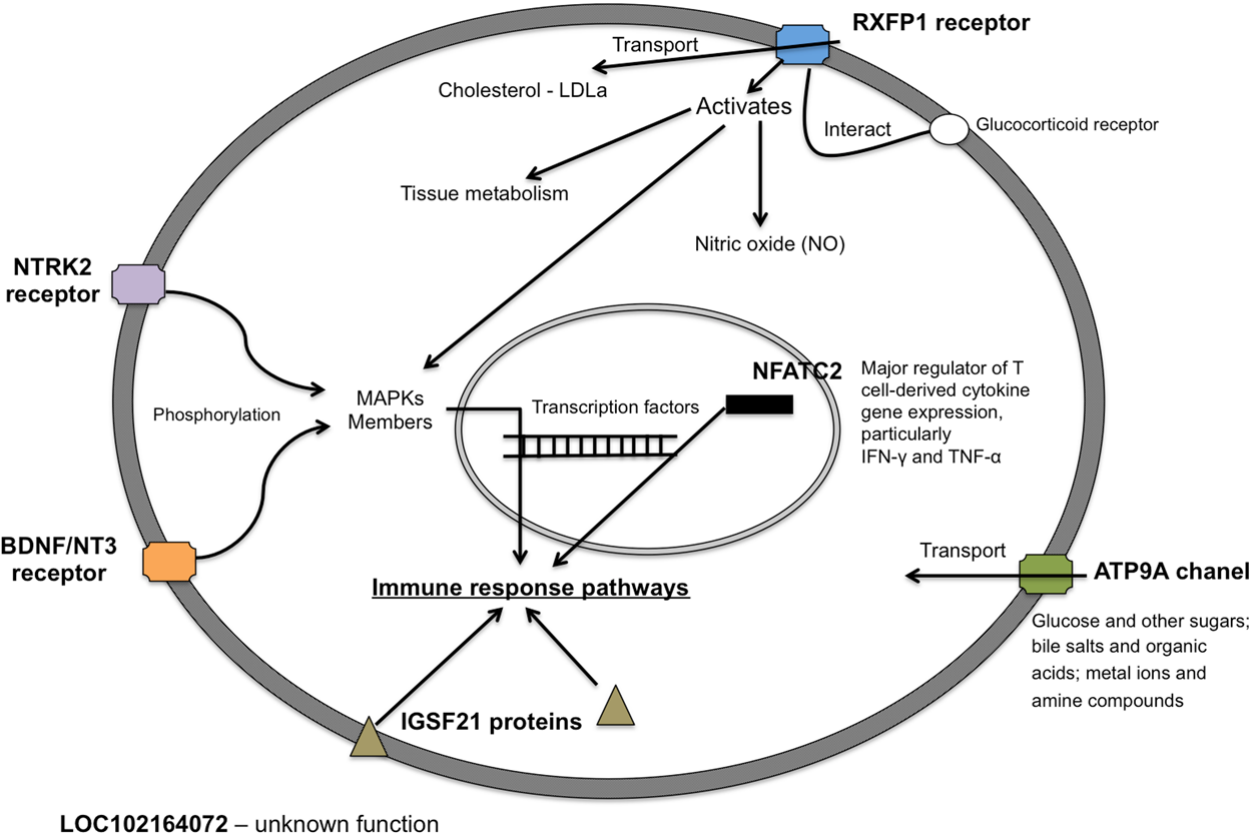
Biological function of genes associated to SNPs with the highest allele frequency differences in the standard and/or stratified genome-wide analyses (GWAS).

**Figure 6:**
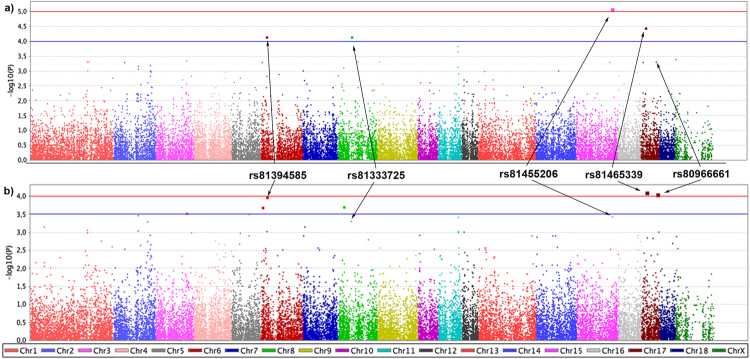
Manhattan plot displaying the genome-wide results [−log10(P)] of the standard (**a**) and stratified (**b**) association analyses between the 2002/06 and 2009/12 time-periods.

The five SNPs (rs81465339, rs81455206, rs81333725, rs81394585 and rs80966661) with the highest divergent allelic frequencies (lower *p-values*) between time-periods (2002/06 *vs*. 2009/12) were initially selected from the standard and stratified case-control analyses (Fig. 6). When these SNPs were validated in a larger dataset, the rs814665339, rs81394585 and rs80966661 SNPs displayed a *p-value* < 6.25 × 10^−3^ (Table 1). By combining the *p-values* of GWAS and validation test, the rs814665339 and rs81394585 SNPs had a *p-value* below the considered threshold of significance (*p* < 1.69E-06). The rs81465339 SNP had the highest allele frequency difference, with the variant (A) being associated with lower odds (OR = 0.123–0.128) of belonging to 2002/06, period with lowest TB prevalence (Table 1). This SNP is closely flanked by *LOC102164072* gene for which there is no information about its biological function (Fig. 5). On the other hand, the variant (A) of rs81394585 SNP, located near to CDH8, was associated with lower odds of belonging to 2002/06 (OR = 0.168–0.170) (Table 1). Finally, and despite no significant result was found in the combined tests, the variant (A) of the rs80966661 SNP, which is located within the *ATP9A* and near to NFATC2 genes, was associated with lower odds of belonging to 2002/06 (OR = 0.081–0.107). The mRNA expression levels varied significantly between time-periods for *LOC102164072 and ATP9A* genes (Table 2). *LOC102164072* had higher levels of mRNA (up-regulated) during 2002/06 (period of lower TB prevalence) when compared with 2009/12, while the *ATP9A* gene had the reverse pattern (down-regulated in 2002/06 in comparison with 2009/12). Detailed analysis of gene expression by age class and time-period/TB outcome revealed different patterns (Fig. S5 and Tables S3, S4 and S5). While *LOC102164072* gene showed significant differences between time-periods (up-regulated in 2002/06) for adults, the expression levels of *ATP9A* varied only for MTC infected adults (down-regulated in 2002/2006). Although no significant results were observed for rs81333725 SNP in the validation and combined tests, the levels of expression of the closest gene *RXFP1* were higher during 2002/06 (period of lower TB prevalence) than 2009/12 (Table 2). The variant (C) of this SNP was associated with lower odds of belonging to 2002/06 (OR = 0.11, 95% CI: 0.03–0.45). Indeed, the variant (C) was significantly associated with a higher level of gene expression (mean = 1.057, 95%CI = 0.792–1.322) when compared with variant (A) (mean = 0.518, 95% CI = 0.349–0.687) (Table S2).

## Discussion

TB is an important threat to wildlife conservation, animal production and human health worldwide. Recent advances in understanding the genetic basis of host susceptibility to TB have revealed several gene variants associated with increased genetic susceptibility in humans^28^ (i.e., ASAP1) and in cattle^17–19^ (i.e., *PTPRT*, *MYO3B* and *SLC6A6*). In this study, we have conducted the first genome-wide screening of genetic variation in a wild reservoir of MTC infection across a temporal scale of increasing TB prevalence, providing new insights into the identification of SNPs (i.e. rs81465339, rs81394585, rs81423166) and candidate genes (i.e. *LOC102164072*, *BDNF/NT-3, NTRK2, CDH8, IGSF21*) associated with genetic susceptibility to TB in the Eurasian wild boar. Our findings also highlighted the putative role of extreme demographic events in shaping host genetic variation in natural populations, and how genetic drift derived from population bottlenecks may affect specific regions of the genome involved in immune response pathways.

Throughout 20 years of wild boar survey, this population underwent three unexplained population crashes (Fig. 1). Fluctuating population sizes have been considered the most important driver of effective population size (N_e_) in natural populations^43^. Successive population crashes (i.e. bottlenecks) may leave a lasting genetic legacy, even if a population subsequently recovers its former abundance, because recovering occurs from a reduced number of individuals at each time^43^. Our predictions using genomic data suggested a continuous decline of N_e_ over the past generations (Fig. 2), which might have resulted from accumulated population bottlenecks that wild boar have been experiencing during the last centuries in the Iberian Peninsula^44^. In particular, the 2004/05 population crash occurred during one of the lowest rainfall periods observed in Mediterranean Spain over the last 50 years^45^. This might have imposed severe life constrains on the wild boar (e.g. reduced food and water resources), and may in turn have led to a high mortality of individuals owing to starvation^37^ and TB^36^. Thereafter, the population recovered to the point at which it reached its maximum abundance in 2010/2011. Although high population abundance represents a risk factor for MTC infection and disease progression^32,39^ and therefore TB-driven selection^36^, the possible increase of genetic drift during the 2004/05 demographic event may also have had an impact on several genomic regions that interfered with the host’s ability to express certain gene variants directly involved in the immune response mechanisms such as *IGSF21*^46^, *BDNF/NT3*^47^ and *NTRK2*^47^ (Fig. 5). In fact, the *IGSF21* gene encodes a protein containing two immunoglobulin (Ig) domains and which is a member of the immunoglobulin superfamily (Fig. 5). Proteins in this superfamily are usually found on or in cell membranes and act as receptors in immune response pathways^46^ (Fig. 5). Furthermore, *BDNF/NT3* and *NTRK2* encode members of the neurotrophic tyrosine receptor kinase family. These kinases are membrane-bound receptors that, upon neurotrophin binding, phosphorylate themselves and members of the MAPK pathway. Pathogenic mycobacteria such as *M. tuberculosis* have evolved mechanisms with which to suppress these signal transduction cascades (i.e. by inhibiting the activation of p38 and ERK1/2) in macrophages and thereby attenuate the production of pro-inflammatory cytokines that induce an immune response (i.e. IL-1, TNF-α and IL-12)^47^.

The source of individuals either from the lowest (2002/06) or highest (2009/12) periods of TB prevalence had a great impact on the GWAS models, which was further confirmed in the validation test (Table 1) and by significantly different mRNA expression levels in individuals from different time-periods (Table 2). However, different patterns of mRNA gene expression were observed according to individual TB phenotype and age class (Fig. S5). This suggests that these candidate genes may confer distinct levels of protection against MTC infection. Some of them also vary according to individual age. For instance, the mRNA expression analyses of *IGSF21* revealed statistically significant differences between time-periods only for the group of adults. On the other hand, although *NFATC2 gene* was not differentially expressed on mRNA analysis, it is a major regulator of the T cell-derived cytokine gene expression, particularly as a key regulator of interferon-γ (IFN-γ) and tumor necrosis factor-α (TNF-α) transcription^48,49^, two of the mechanisms used by pathogenic mycobacteria to survive and persist in host macrophages^48–51^ (Fig. 5). The age class (juvenile or adult) does not, however, appear to influence the GWAS models. This supports previous evidence suggesting that MTC infection already takes place during the first months of life, which in turn imposes an early selective pressure on wild boar^34^.

Analyses of the mRNA expression profile of the *MUT* and *C3* genes, two loci that have been previously reported to be involved in wild boar genetic susceptibility to TB^24,52^, showed that there were statistically significant differences between time-periods but not between TB outcomes. Further analyses by age class and host TB phenotype revealed statistically significant differences between time-periods only for the group of infected adults. The high number of individuals with disseminated TB lesions in 2009/12 might explain this outcome, although they were randomly sampled in both periods, suggesting some level of increased genetic susceptibility to MTC infection over time. The up-regulation of the *MUT* gene in mandibular lymph nodes and tonsils has been associated with a protective response to MTC infection in uninfected individuals^24,40,53^. Our findings suggested that *MUT* could also be involved in the genetic mechanisms that determine the dissemination of TB lesions throughout the organism, as evidenced by the higher expression levels observed in 2002/06 in comparison with 2009/12^53^. Furthermore, the *C3* gene revealed the reverse pattern. The higher expression levels recorded in 2009/12 when compared with 2002/06 suggest that infected individuals with disseminated TB lesions may express higher *C3* levels in an attempt to control infection. This is in agreement with recent findings that showed an increase in *C3* mRNA levels in response to mycobacterial infection^54^, and, therefore, its important role in protection against TB^55^.

In summary, in this study we have used the unique Iberian context regarding TB in wild boar to provide new insights into host-pathogen co-evolution in natural populations. In addition to being one of the few known wildlife reservoirs of TB^1,32^, the wild boar is the ancestor of the domestic pig, one of the most important model species used in human infectious diseases because of its physiology and immune response similarity with humans^56,57^. Despite all the constraints related to host disease characterization in natural populations (e.g. estimating contact rates between host and pathogen and time of infection within the host system) and the absence of high number of genomic data in pig and wild boar for performing a fine-mapping imputation, we were able to identify several coding genes as candidates for wild boar genetic susceptibility to TB. In addition, our findings highlighted the putative role of demographic events in shaping genomic variation in natural populations and how they may impact on particular regions of the genome that affect the host immune response to infections. These findings have important implications for wildlife populations because one of the few available disease control measures in nature is through the management of the host, which usually involves population density reduction^58^. Population bottlenecks may lead to changes in specific genomic regions that interfere with the natural co-evolution of host and pathogens and thus disrupt the disease-mediated selection that occurs in nature. And finally, the candidate genes provided herein advanced our understanding of genetic mechanisms underlying host genetic susceptibility to TB, and may represent important genetic tools for future research in a larger number of host species, including humans.

## Materials and Methods

### Ethics statement

All animal sampling took place post-mortem. The wildlife samples were obtained from hunter-harvested individuals that were shot during control programs implemented in a nature reserve and independently and prior to our research. According to EU and National legislation (2010/63/UE Directive and Spanish Royal Decree 53/2013) and to the University of Castilla—La Mancha guidelines, no permission or consent is required to conduct the research reported herein.

### Study area

The study was carried out in a nature reserve located in the central-southern region of Spain, “Los Quintos de Mora” (30 S: 408219E, 4363199 N). The habitat is Mediterranean and characterized by evergreen oak *Quercus ilex* woodlands and scrublands (mainly *Cystus* spp, *Erica* spp, *Pistacia* spp, *Phyllirea* spp and *Rosmarinus* spp), with scattered pastures and small crops, known as “dehesa”^39^. These savannah-like landscapes are highly seasonal as regards natural resources, with constraints for autochthonous ungulates (i.e. wild boar, red deer and roe deer) mostly during the hot dry season. The reserve extends over 6.864 hectares and is characterized by an extensive flat area surrounded by two mountains. The implementation of a monitoring program in this reserve since 1993 allowed us to follow the number, sex and age class of the majority of animals culled. This data was used as a proxy of population abundance, since an identical procedure has been constantly applied throughout the last 20 years (Fig. 1 and Table S1). Population control was based on hunting with the assistance of small packs of dogs (i.e. “batidas”), which extracted individuals in a random manner.

### Sampling and host TB phenotype

Wild boar sampling was carried out during ten consecutive seasons (September to August), from 2002–03 to 2011–12. In each hunting season, a set (range 3–125) of individuals was culled and necropsied in the field. In addition to the individual characterization regularly recorded in the reserve (i.e. sex, age class), a detailed macroscopical inspection followed by a collection of various TB target tissues and organs was also performed^39^. Once in the laboratory, the tissues and organs were serially dissected, sectioned and carefully examined for the presence/absence of TB-like lesions^35^. A pool of sampled tissues/organs was used to confirm the presence/absence of MTC infection by means of mycobacterial culture^32,39^. These tests, when used in parallel, have proven to have a 95.5% sensitivity and a 100% specificity as regards wild boar TB^59^. Using the retrospective results of the TB culture, the age of the animals and the time-periods considered in our hypotheses (2002/06 vs 2009/12) as a basis, a total of 100 individuals were selected for whole genome characterization (Table 3). Infected individuals therefore had macroscopic TB-like lesions, a positive TB culture or both, while uninfected animals were those with an absence of TB-like lesions in gross pathology and negative result in the mycobacterial culture. The adults were further classified according to the level of disseminated TB-like lesions: (i) individuals with restricted lesions in mandibular lymph nodes; and (ii) individuals with generalized lesions (i.e. presence of lesions in more than one tissue/organs).

**Table 3:** Sampled wild boar used throughout this study by time-period, tuberculosis (TB) outcome and age class: (i) number of individuals genotyped with the Illumina porcine SNP60 BeadChips; (ii) number of individuals included in the genome-wide association (GWAS); (iii) number of individuals used to validate significant SNPs identified in GWAS.

Time-period	Tuberculosis (TB)			Age
	Uninfected	Infected		
		Restricted TB-like lesions	Generalized TB-like lesions	
**2002/06**	i) 8, II) 4, iii) 8	i) 5, ii) 0, iii) 2		**Juvenile**
	i) 4, ii) 2, iii) 3	i) 6, ii) 3, iii) 5	i) 4, ii) 0, iii) 2	**Adult**
**2009/12**	i) 12, ii) 6, iii) 11	i) 21, ii) 8, iii) 15		**Juvenile**
	i) 7, ii) 4, iii) 6	i) 13, ii) 7, iii) 9	i) 20, ii) 10, iii) 15	**Adult**
**TOTAL**	i) 20, ii) 10, iii) 19	i) 26, ii) 8, iii) 17		**Juvenile**
	i) 11, ii) 6, iii) 9	i) 19, ii) 10, iii) 14	i) 24, ii) 10, iii) 17	**Adult**
	i) 100, ii) 44, iii) 76			**All**

The level of disseminated TB-like lesions was determined only for adult individuals.

### Population demographic history

In order to confirm that the population studied represented a proper natural population, with an absence of human-induced effects such as hybridization with commercial and Iberian pig breeds or even restocking of wild boar from central and northern Europe, we assessed the level of population differentiation with regard to data published on domestic pigs and wild boar^60,61^. We used a subset of common SNPs to conduct a principal component analysis (PCA) with the adgenet software^62^, and a Bayesian clustering approach in the STRUCTURE program^63^. Default STRUCTURE parameters were set together with an admixture model in combination with correlated allele frequencies^64^ and no prior-information about population origin. The log likelihood of the data [ln Pr(*X*/*K*)] was calculated for *K* = 1 to *K* = 6 with 2 repetitions of 10^6^ MCMC iterations following a burn-in period of 10^5^ steps. Moreover, Δ*K* was calculated by following^65^ and using STRUCTURE HARVESTER^66^. PCA and STRUCTURE analyses were conducted using 15437 SNPs genotyped in 313 individuals. This dataset was achieved: after pruning the SNPs with the genotyping rate and a minor allele frequency lower than 95%; SNPs not in the Hardy-Weinberg equilibrium (*p* < 0.0001); SNPs in linkage disequilibrium (r^2^ > 0.5) in windows of 50 SNPs and shift 5 SNPs forward between each window (indep-paiwise 50 5 0.5); and finally the SNPs of sexual chromosomes. Filtering analyses were carried out in PLINK 1.9^67^. Additionally, in order to ensure the absence of a population substructure within our sampled population we constructed another PCA using the sampled wild boar clustered by time-period and TB outcome, and estimated the levels of differentiation using PLINK 1.9^67^.

In order to address the historical effective population size (N_e_) trajectories throughout the past generations, we applied the linkage disequilibrium method implemented with SNeP V1.1 software following the author’s recommendations^68^. The generation time assumed for wild boar was an average of one year^69,70^.

### DNA/RNA extraction and genotyping

Genomic DNA and total RNA were extracted from the mandibular lymph nodes using the AllPrep DNA/RNA/Protein Mini Kit (QUIAGEN) and following the manufacture’s recommendations. RNA was stored at −80 °C until subsequent mRNA expression analysis. Individuals were genotyped using the Illumina porcine SNP60 BeadChips^71^, from which 61565 SNPs were obtained. The average genotyping rate was 0.92, varying across individuals from 0.50 to 0.99.

### Genome-wide quality control analysis

A rigorous check of the initial dataset was carried out by using PLINK 1.9^67^ and following the quality control steps widely recommended for GWAS^72,73^. A total of 29504 SNPs genotyped in 44 individuals (genotyping rate of 0.998) achieved the recommended standard values^72,73^. This amount of SNPs and individuals was obtained after filtering the initial data as follows: I) SNPs with duplicated (rs) mapping name (*n* = 21) on current pig genome assembly (Sscrofa10.2), monomorphic SNPs (*n* = 25242), SNPs with a GentrainScore lower than 0.7 (*n* = 1592), SNPs with an allele frequency below 0.05 (*n* = 4604), SNPs with a genotyping rate lower than 95% (*n* = 587) and SNPs not in the Hardy-Weinberg equilibrium (in controls, *n* = 15); II) individuals with a genotyping rate lower than 95% (*n* = 32), individuals with heterozygosity rates in X-chromosomes, SNPs outside the boundaries of sex assignment (*n* = 4) and individuals with more than 0.185 of identity-by-descent (*n* = 20).

### Genome-wide associations (GWAS) and validation test

Two complementary genome-wide associations (GWAS) were conducted in this study to find segments of genome (i.e. carrying candidate genes) possibly related to an increased genetic susceptibility of wild boar to TB: *(i)* a classical case-control analysis, during which individuals infected with MTC were compared with those that were uninfected, and *(ii)* a case-control analysis of individuals from the lowest and highest periods of TB prevalence (2002/06 *vs*.2009/12). In this case, we aimed to investigate whether the huge increase in TB prevalence was accomplished by changes in particular regions of genome (Fig. S6).

Owing to the limited sample size, genome-wide associations (case-control analyses) were constructed in a simplistic manner with PLINK1.9, using both the standard and stratified analyses^67^. The standard association was applied because recent evidence has suggested that MTC infection affects individuals from younger ages and might cause high mortality^34,36^. Furthermore, we applied the stratified analyses (Cochran-Mantel-Haenszel tests^67^) to account for age class and time-period/TB outcome in the statistical models, which allowed us to compute a weighted average of the per-stratum odds ratios for each SNP variant. Given the controversy for defining a statistically significant result in GWAS^72,73^, we assumed as initial step an empirical cut-off in the *p-values* distribution to select the highest differentiated SNPs, since none of the SNPs would remain significant whether a conservative Bonferroni correction method was applied (*p-values* < 1.69E-06). The considered threshold (*p-value* < 1.00E-4), which represent the top 0.03% of the lowest *p-value* results obtained in GWAS, allowed the selection of eight SNPs, which were further validated in a larger dataset that incorporated all the individuals with a genotyping rate higher than 95%. This procedure increased the initial dataset of 44 individuals (GWAS_Season_: *n* = 9 in 2002/06 and *n* = 36 in 2009/12; GWAS_TB_: *n* = 16 uninfected and *n* = 28 infected) to 76 individuals (GWAS_Season_: *n* = 20 in 2002/06 and *n* = 56 in 2009/12; GWAS_TB_: *n* = 28 uninfected and *n* = 48 infected). The *p-values* and OR of GWAS and validation tests were also combined using the meta-analysis function incorporated in PLINK1.9^67^, and the initial conservative *p-value* of 1.69E-06 was assumed as a threshold of significance. In addition to this, the genome inflation factor, QQ-plots and the statistical power of the GWAS were calculated. Power and sample size calculations were performed with a web-browser program, GENETIC POWER CALCULATOR^74^. We assumed a disease prevalence of 45% (initially recorded, complete linkage disequilibrium (D’ = 1), 5% type I error rate (α) and a relative risk associated to minor allele frequency (MAF) genotype of 0.01. We computed power and size sample calculations as a function of high-risk allele frequency (0.05, 0.10 and 0.15) for both GWAS (TB outcome and Time-period) and according to the respective control-case ratio of each analysis (Table S6). Outputs from these analyses indicated that there was more than 80% power of detecting loci at *p* < 0.05 with high-risk allele frequency above 0.10 in both GWAS analyses.

The porcine genome assembly 10.2 (http://www.ensembl.org/Sus_scrofa/Info/Index) and National Center for Biotechnology Information (NCBI) Genome (http://www.ncbi.nlm.nih.gov/genome/?term=pig) were retrieved to characterize the genomic regions around the significant SNPs identified on GWAS. Function and signal pathway of the selected candidate genes were found via Ensembl Biomart (http://www.biomart.org/), GeneCards (http://www.genecards.org/), NCBI Gene (http://www.ncbi.nlm.nih.gov/gene/).

### mRNA expression profile

In order to assess whether the statistically significant SNPs identified in various chromosome segments are in linkage disequilibrium with close genes, we selected seven candidate genes (i.e. preferably those related to immune response) to quantify the mRNA expression levels knowing that transcriptome might vary throughout the developmental stage of disease^75^ and post-translation processes may also occur after transcription^76^. Oligonucleotide primers were designed for each candidate gene using the NCBI Primer-Blast tool^47^ (Table 4). A semi-quantitative real-time RT-PCR was performed using the One-Step RT-PCR Kit with SYBR Green and the CFX thermal cycler (Bio-Rad, Hercules, CA, USA) following the manufacturer’s instructions. A dissociation curve was run at the end of RT-PCR reaction to ensure that only one amplicon was formed and that the amplicon denatured consistently at the same temperature range for every sample^77^. When the size of RT-PCR products did not correspond to the predicted size on Primer-Blast (i.e. confirmed by electrophoresis in agar gel) some samples were randomly selected for sequencing. *NTRK2* was the only gene that had a different fragment length. After sequencing analysis and a later annotation on a *S. scrofa* 10.2 assembly (Annotation Release 105), this fragment was allocated to chromosome 18 (location 38,299,366 to 38,385,443 bp), between two uncharacterized genes (*LOC102167850* and *LOC102167929*). The mRNA expression profiles of the MUT and C3 genes, two genes that had different expression levels in infected and uninfected individuals with MTC^24,40,53^, were also characterized. The mRNA expression values were normalized against *S. scrofa* cyclophilin, b-actin and H3FSA using the genNorm ddCT method^78^. Since the 44 individuals included in the genomic analysis were not equally distributed across periods (*n* = 9 in 2002/06 *vs. n* = 35 in 2009/12), the mRNA expression levels were also quantified for the remaining 18 individuals from the 2002/06 time-period. However, it was not possible to determine Ct values for some genes in various individuals. This may have been owing to mRNA degradation and/or mutations on the primer binding sites. It is important to note that at least four combinations of primers sets were tested in the case of these genes. The levels of mRNA expression (normalized Ct values) were therefore compared between time-periods (2002/06 *vs*. 2009/12) and TB outcomes (infected *vs*. uninfected) using the Student’s t-test with unequal variance (*p-value* = 0.05). This test was also applied to compare the expression values between age class and time-period/TB outcome. Generalized linear models (GLMs) were conducted to assess the relationship between SNP alleles and expression levels of the closest genes. The age class and time-period/TB outcome were incorporated in these models in order to account for their possible effects. The models were run in SPSS, version 20 (IBM Analytics).

**Table 4:** RT-PCR conditions for the candidate genes explored in the mRNA expression analyses.

Analysis	Gene—GenBank		Primer sequences (5′–3′)	RT-PCR	
	Name	Chr		Expected size (bp)	Annealing conditions
2002/06 *vs*. 2009/12	LOC102164072	17	F = TCTGGCTGTGTCAACCAACA R = CGTCAGAAGCATGCACTCCA	93	60 °C, 30 s
	RXFP1	8	F = CCTGGTACCTCGTTATCCCC R = AGACATTGCATGGGCACAGA	75	60 °C, 30 s
	ATP9A	17	F = CCATGCAGGCTGTCTTTTCC R = ATCCTTGTAGAGCTCGGGGT	173	60 °C, 30 s
	NFATC2		F = GGGCAGCAGATTTGGGAGAT R = GCTGTGGGTAGTAAGGCTGG	270	60 °C, 30 s
Uninfected *vs*. Infected	LOC100621290 BDNF/NT-3	10	F = AGAGGGTGTTTGAGGGGAAG R = AGCACCGTCCCTTGTGAAAT	78	60 °C, 30 s
	NTRK2		F = TCTCGGTCTACGCTGTGGTA R = AGCTGTTCCGACTAACGGTC	192	54 °C, 30 s
	IGSF21	6	F = CGACACCAAGATGCAGAGGT R = CGTCTCGGGGATGTTCTCAG	142	60 °C, 30 s

### Data deposition

Raw SNP data, all managed datasets and GWAS summary statistics were deposited in a dryad repository DOI:10.5061/dryad.39c3k (temporary link: http://datadryad.org/review?doi=doi:10.5061/dryad.39c3k).

## Electronic supplementary material

Supplementary Information
