# Supplementary material for: Genome-wide associations identify novel candidate loci associated with genetic susceptibility to tuberculosis in wild boar
Source: Sci Rep. 2018 Jan 31;8:1980. doi: 10.1038/s41598-018-20158-x (PMC5792637; doi:10.1038/s41598-018-20158-x)
Supplement: Supplementary file 1 — Supplementary Information [file 41598_2018_20158_MOESM1_ESM.doc]

**Title:** Genome-wide associations identify novel candidate loci associated with genetic susceptibility to tuberculosis in wild boar

**Authors**: João Queirós^1,2,3, #^, Paulo Célio Alves^1,2,4^, Joaquín Vicente^3^, Christian Gortazar^3^, José de la Fuente^3,5^

**Affiliation**:

1 - Centro de Investigacão em Biodiversidade e Recursos Genéticos (CIBIO)/InBio Laboratório Associado, Universidade do Porto, Campus Agrário de Vairão, R. Monte-Crasto, 4485-661 Vairão, Portugal.

2 - Departamento de Biologia, Faculdade de Ciências da Universidade do Porto (FCUP), Rua do Campo Alegre s⁄n, 4169-007 Porto, Portugal.

3 - SaBio, Instituto de Investigación en Recursos Cinegéticos IREC (CSIC-UCLM-JCCM), Ronda de Toledo s/n, 13071 Ciudad Real, Spain.

4 - Wildlife Biology Program, University of Montana, Missoula, MT 59812, USA.

5 -Department of Veterinary Pathobiology, Center for Veterinary Health Sciences, Oklahoma State University, Stillwater, OK 74078, USA.

E-mail addresses: [joaoluis.teixeira@uclm.es](mailto:jJoaolLuis.tTeixeira@uclm.es)

[pcalves@fc.up.pt](mailto:pcalves@fc.up.pt)

[joaquin.vicente@uclm.es](mailto:joaquin.vicente@uclm.es)

[christian.gortazar@uclm.es](mailto:christian.gortazar@uclm.es)

[jose_delafuente@yahoo.com](mailto:jose_delafuente@yahoo.com)

# - Correspondence to [joaoluis.teixeira@uclm.es](mailto:jJoaolLuis.tTeixeira@uclm.es)

SaBio, Instituto de Investigación en Recursos Cinegéticos IREC (CSIC-UCLM-JCCM), Ronda de Toledo s/n, 13071 Ciudad Real, Spain. Tel.: 0034 926 295 450 / 926 295 300 Fax: 926 295 451

**Table S1:** Age class and sex of individuals culled during the control programs implemented in the reserve.

**Table S2: Results of the generalized linear models (GLMs) conducted to assess the association between SNP alleles and mRNA expression of the closest genes.**

| **Candidate genes** | **Variables** | **Wald Chi-square** | **Degrees freedom** | ***p-value*** | **SNP variant** | **B estimate** | **Mean** | **95% CI** | |
| --- | --- | --- | --- | --- | --- | --- | --- | --- | --- |
|  |  |  |  |  |  |  |  | **Lower** | **Upper** |
| **BDNT/NT-3** | Intercept | 119.79 | 1 | 0.000 |  |  |  |  |  |
|  | Sex | 1.70 | 1 | 0.193 |  |  |  |  |  |
|  | TB | 32.78 | 1 | 0.000 |  |  |  |  |  |
|  | rs81423166 | 0.05 | 1 | 0.832 | A | 0.012 | 0.371 | 0.301 | 0.441 |
|  |  |  |  |  | G | 0* | 0.359 | 0.261 | 0.457 |
| **IGSF21** | Intercept | 83.56 | 1 | 0.000 |  |  |  |  |  |
|  | Sex | 5.33 | 1 | 0.021 |  |  |  |  |  |
|  | TB | 18.44 | 1 | 0.000 |  |  |  |  |  |
|  | **rs81388748** | **4.08** | **1** | **0.044** | **A** | **-0.122** | **0.270** | **0.172** | **0.368** |
|  |  |  |  |  | **C** | **0*** | **0.392** | **0.305** | **0.479** |
| **Loc102164072** | Intercept | 78.58 | 1 | 0.000 |  |  |  |  |  |
|  | Sex | 0.38 | 1 | 0.540 |  |  |  |  |  |
|  | TB | 3.01 | 1 | 0.083 |  |  |  |  |  |
|  | Rs81465339 | 3.25 | 1 | 0.072 | A | -0.159 | 0.328 | 0.223 | 0.433 |
|  |  |  |  |  | G | 0* | 0.487 | 0.345 | 0.629 |
| **RXFP1** | Intercept | 95.63 | 1 | 0.000 |  |  |  |  |  |
|  | Sex | 0.64 | 1 | 0.423 |  |  |  |  |  |
|  | TB | 1.31 | 1 | 0.253 |  |  |  |  |  |
|  | **rs81333725** | **11.46** | **1** | **0.001** | **A** | **-0.539** | **0.518** | **0.349** | **0.687** |
|  |  |  |  |  | **C** | **0*** | **1.057** | **0.792** | **1.322** |
| **NFATC** | Intercept | 31.68 | 1 | 0.000 |  |  |  |  |  |
|  | Sex | 0.10 | 1 | 0.747 |  |  |  |  |  |
|  | TB | 1.02 | 1 | 0.312 |  |  |  |  |  |
|  | rs80966661 | 0.00 | 1 | 0.998 | A | -0.0001 | 0.0758 | 0.0273 | 0.1243 |
|  |  |  |  |  | G | 0* | 0.0759 | 0.0579 | 0.0938 |
| **ATP9A** | Intercept | 10.678 | 1 | 0.001 |  |  |  |  |  |
|  | Sex | 0.012 | 1 | 0.911 |  |  |  |  |  |
|  | TB | 0.014 | 1 | 0.905 |  |  |  |  |  |
|  | rs80966661 | 0.023 | 1 | 0.878 | A | 0.002 | 0.025 | -0.002 | 0.052 |
|  |  |  |  |  | G | 0* | 0.023 | 0.016 | 0.030 |

*, Zero represents a referent value for comparison.

**Table S3**: Expression profile (normalized Ct values) of genes associated to SNPs with the highest allele frequency differences in the standard and/or stratified genome-wide analyses (GWAS). Differences between time-periods were quantified for uninfected and infected individuals with *Mycobacterium tuberculosis complex* (MTC) and for juvenile and adults animals using an independent t-test.

| **Gene** | **Tuberculosis** | **Age class** | **Time-period** | **N** | **Mean** | **SE** | **t** | **df** | ***p-value*** |
| --- | --- | --- | --- | --- | --- | --- | --- | --- | --- |
| **LOC102164072** | Non-tuberculous | Juvenile | 2002/06 | 7 | 0.56 | 0.052 | 0.8 | 6.5 | 0.476 |
|  |  |  | 2009/12 | 6 | 0.45 | 0.134 |  |  |  |
|  |  | Adults | 2002/06 | 4 | 0.66 | 0.102 | 3.9 | 5.2 | 0.010 |
|  |  |  | 2009/12 | 4 | 0.18 | 0.067 |  |  |  |
|  | Tuberculous | Juvenile | 2002/06 | 3 | 0.57 | 0.142 | 1.1 | 4.5 | 0.314 |
|  |  |  | 2009/12 | 8 | 0.37 | 0.106 |  |  |  |
|  |  | Adults | 2002/06 | 9 | 0.68 | 0.093 | 4.1 | 14.9 | 0.001 |
|  |  |  | 2009/12 | 17 | 0.22 | 0.061 |  |  |  |
| **RXFP1** | Non-tuberculous | Juvenile | 2002/06 | 8 | 0.79 | 0.078 | -0.2 | 5.9 | 0.811 |
|  |  |  | 2009/12 | 6 | 0.86 | 0.261 |  |  |  |
|  |  | Adults | 2002/06 | 4 | 0.85 | 0.239 | 1.7 | 5.1 | 0.149 |
|  |  |  | 2009/12 | 4 | 0.37 | 0.153 |  |  |  |
|  | Tuberculous | Juvenile | 2002/06 | 4 | 0.65 | 0.114 | 0.5 | 9.7 | 0.652 |
|  |  |  | 2009/12 | 8 | 0.56 | 0.148 |  |  |  |
|  |  | Adults | 2002/06 | 10 | 1.07 | 0.165 | 3.6 | 15.3 | 0.002 |
|  |  |  | 2009/12 | 17 | 0.38 | 0.097 |  |  |  |
| **ATP9A** | Non-tuberculous | Juvenile | 2002/06 | 0 |  |  |  |  |  |
|  |  |  | 2009/12 | 5 | 0.02 | 0.008 |  |  |  |
|  |  | Adults | 2002/06 | 1 | 0.00 |  |  |  |  |
|  |  |  | 2009/12 | 4 | 0.03 | 0.013 |  |  |  |
|  | Tuberculous | Juvenile | 2002/06 | 0 |  |  |  |  |  |
|  |  |  | 2009/12 | 8 | 0.03 | 0.009 |  |  |  |
|  |  | Adults | 2002/06 | 3 | 0.00 | 0.000 | -5.8 | 16.1 | 0.000 |
|  |  |  | 2009/12 | 17 | 0.02 | 0.004 |  |  |  |
| **NFATC** | Non-tuberculous | Juvenile | 2002/06 | 2 | 0.04 | 0.017 | -0.7 | 5.8 | 0.515 |
|  |  |  | 2009/12 | 6 | 0.06 | 0.031 |  |  |  |
|  |  | Adults | 2002/06 | 2 | 0.08 | 0.039 | -0.1 | 1.3 | 0.950 |
|  |  |  | 2009/12 | 4 | 0.08 | 0.014 |  |  |  |
|  | Tuberculous | Juvenile | 2002/06 | 0 |  |  |  |  |  |
|  |  |  | 2009/12 | 8 | 0.06 | 0.018 |  |  |  |
|  |  | Adults | 2002/06 | 3 | 0.11 | 0.036 | 0.5 | 2.6 | 0.639 |
|  |  |  | 2009/12 | 17 | 0.09 | 0.014 |  |  |  |
| **BDNF/NT3** | Non-tuberculous | Juvenile | 2002/06 | 8 | 0.70 | 0.097 | 2.7 | 11.7 | 0.019 |
|  |  |  | 2009/12 | 6 | 0.33 | 0.096 |  |  |  |
|  |  | Adults | 2002/06 | 4 | 0.68 | 0.129 | 4.0 | 3.9 | 0.017 |
|  |  |  | 2009/12 | 4 | 0.12 | 0.049 |  |  |  |
|  | Tuberculous | Juvenile | 2002/06 | 4 | 0.89 | 0.182 | 3.4 | 3.9 | 0.029 |
|  |  |  | 2009/12 | 8 | 0.23 | 0.069 |  |  |  |
|  |  | Adults | 2002/06 | 10 | 1.06 | 0.114 | 7.5 | 11.1 | 0.000 |
|  |  |  | 2009/12 | 17 | 0.15 | 0.039 |  |  |  |
| **IGSF21** | Non-tuberculous | Juvenile | 2002/06 | 8 | 0.55 | 0.065 | 1.6 | 8.5 | 0.151 |
|  |  |  | 2009/12 | 6 | 0.36 | 0.107 |  |  |  |
|  |  | Adults | 2002/06 | 4 | 0.43 | 0.064 | 4.2 | 5.7 | 0.006 |
|  |  |  | 2009/12 | 4 | 0.09 | 0.051 |  |  |  |
|  | Tuberculous | Juvenile | 2002/06 | 4 | 0.66 | 0.193 | 1.9 | 4.7 | 0.118 |
|  |  |  | 2009/12 | 8 | 0.24 | 0.100 |  |  |  |
|  |  | Adults | 2002/06 | 10 | 0.60 | 0.069 | 6.2 | 14.1 | 0.000 |
|  |  |  | 2009/12 | 17 | 0.12 | 0.036 |  |  |  |
| **NTRK** | Non-tuberculous | Juvenile | 2002/06 | 7 | 0.02 | 0.006 | 1.3 | 10.9 | 0.228 |
|  |  |  | 2009/12 | 6 | 0.01 | 0.006 |  |  |  |
|  |  | Adults | 2002/06 | 4 | 0.01 | 0.006 | 1.5 | 3.9 | 0.204 |
|  |  |  | 2009/12 | 4 | 0.00 | 0.002 |  |  |  |
|  | Tuberculous | Juvenile | 2002/06 | 3 | 0.01 | 0.005 | 0.3 | 7.2 | 0.753 |
|  |  |  | 2009/12 | 8 | 0.01 | 0.005 |  |  |  |
|  |  | Adults | 2002/06 | 10 | 0.03 | 0.005 | 4.2 | 16.5 | 0.001 |
|  |  |  | 2009/12 | 15 | 0.01 | 0.003 |  |  |  |
| **MUT** | Non-tuberculous | Juvenile | 2002/06 | 8 | 0.94 | 0.078 | -1.0 | 6.5 | 0.333 |
|  |  |  | 2009/12 | 6 | 1.16 | 0.205 |  |  |  |
|  |  | Adults | 2002/06 | 4 | 1.09 | 0.118 | 2.0 | 5.3 | 0.099 |
|  |  |  | 2009/12 | 4 | 0.67 | 0.173 |  |  |  |
|  | Tuberculous | Juvenile | 2002/06 | 4 | 1.00 | 0.055 | 0.0 | 8.0 | 0.977 |
|  |  |  | 2009/12 | 8 | 1.00 | 0.194 |  |  |  |
|  |  | Adults | 2002/06 | 10 | 1.31 | 0.130 | 4.2 | 11.9 | 0.001 |
|  |  |  | 2009/12 | 17 | 0.72 | 0.051 |  |  |  |
| **C3** | Non-tuberculous | Juvenile | 2002/06 | 4 | 0.00 | 0.000 | -1.6 | 5.0 | 0.174 |
|  |  |  | 2009/12 | 6 | 0.01 | 0.006 |  |  |  |
|  |  | Adults | 2002/06 | 3 | 0.00 | 0.001 | -1.6 | 3.1 | 0.199 |
|  |  |  | 2009/12 | 4 | 0.02 | 0.010 |  |  |  |
|  | Tuberculous | Juvenile | 2002/06 | 3 | 0.00 | 0.000 | -2.9 | 7.0 | 0.023 |
|  |  |  | 2009/12 | 8 | 0.01 | 0.005 |  |  |  |
|  |  | Adults | 2002/06 | 7 | 0.00 | 0.001 | -4.0 | 21.2 | 0.001 |
|  |  |  | 2009/12 | 17 | 0.01 | 0.003 |  |  |  |

**Table S4**: Expression profile (normalized Ct values) of genes associated to SNPs with the highest allele frequency differences in the standard and/or stratified genome-wide analyses (GWAS). Differences between uninfected and infected individuals with *Mycobacterium tuberculosis complex* (MTC) were quantified for 2002/06 and 2009/12 time-periods and for juvenile and adult animals using an independent t-test.

| **Gene** | **Time-period** | **Age class** | **Tuberculosis** | **N** | **Mean** | **SE** | **t** | **df** | ***p-value*** |
| --- | --- | --- | --- | --- | --- | --- | --- | --- | --- |
| **LOC102164072** | 2002/06 | Juvenile | Non-tuberculous | 7 | 0.56 | 0.052 | 0.0 | 2.6 | 0.981 |
|  |  |  | Tuberculous | 3 | 0.57 | 0.142 |  |  |  |
|  |  | Adults | Non-tuberculous | 4 | 0.66 | 0.102 | -0.1 | 8.0 | 0.906 |
|  |  |  | Tuberculous | 9 | 0.68 | 0.093 |  |  |  |
|  | 2009/12 | Juvenile | Non-tuberculous | 6 | 0.45 | 0.134 | 0.5 | 10.4 | 0.615 |
|  |  |  | Tuberculous | 8 | 0.37 | 0.106 |  |  |  |
|  |  | Adults | Non-tuberculous | 4 | 0.18 | 0.067 | -0.4 | 8.8 | 0.690 |
|  |  |  | Tuberculous | 17 | 0.22 | 0.061 |  |  |  |
| **RXFP1** | 2002/06 | Juvenile | Non-tuberculous | 8 | 0.79 | 0.078 | 1.1 | 5.9 | 0.330 |
|  |  |  | Tuberculous | 4 | 0.65 | 0.114 |  |  |  |
|  |  | Adults | Non-tuberculous | 4 | 0.85 | 0.239 | -0.8 | 6.1 | 0.465 |
|  |  |  | Tuberculous | 10 | 1.07 | 0.165 |  |  |  |
|  | 2009/12 | Juvenile | Non-tuberculous | 6 | 0.86 | 0.261 | 1.0 | 8.1 | 0.343 |
|  |  |  | Tuberculous | 8 | 0.56 | 0.148 |  |  |  |
|  |  | Adults | Non-tuberculous | 4 | 0.37 | 0.153 | -0.1 | 5.7 | 0.952 |
|  |  |  | Tuberculous | 17 | 0.38 | 0.097 |  |  |  |
| **ATP9A** | 2002/06 | Juvenile | Non-tuberculous | 0 |  |  |  |  |  |
|  |  |  | Tuberculous | 0 |  |  |  |  |  |
|  |  | Adults | Non-tuberculous | 1 | 0.00 |  |  |  |  |
|  |  |  | Tuberculous | 3 | 0.00 | 0.000 |  |  |  |
|  | 2009/12 | Juvenile | Non-tuberculous | 5 | 0.02 | 0.008 | -0.8 | 10.4 | 0.445 |
|  |  |  | Tuberculous | 8 | 0.03 | 0.009 |  |  |  |
|  |  | Adults | Non-tuberculous | 4 | 0.03 | 0.013 | 0.8 | 3.5 | 0.460 |
|  |  |  | Tuberculous | 17 | 0.02 | 0.004 |  |  |  |
| **NFATC** | 2002/06 | Juvenile | Non-tuberculous | 2 | 0.04 | 0.017 |  |  |  |
|  |  |  | Tuberculous | 0 |  |  |  |  |  |
|  |  | Adults | Non-tuberculous | 2 | 0.08 | 0.039 | -0.6 | 2.5 | 0.617 |
|  |  |  | Tuberculous | 3 | 0.11 | 0.036 |  |  |  |
|  | 2009/12 | Juvenile | Non-tuberculous | 6 | 0.06 | 0.031 | 0.0 | 8.3 | 0.996 |
|  |  |  | Tuberculous | 8 | 0.06 | 0.018 |  |  |  |
|  |  | Adults | Non-tuberculous | 4 | 0.08 | 0.014 | -0.4 | 10.2 | 0.731 |
|  |  |  | Tuberculous | 17 | 0.09 | 0.014 |  |  |  |
| **BDNF/NT3** | 2002/06 | Juvenile | Non-tuberculous | 8 | 0.70 | 0.097 | -0.9 | 4.8 | 0.403 |
|  |  |  | Tuberculous | 4 | 0.89 | 0.182 |  |  |  |
|  |  | Adults | Non-tuberculous | 4 | 0.68 | 0.129 | -2.2 | 7.9 | 0.060 |
|  |  |  | Tuberculous | 10 | 1.06 | 0.114 |  |  |  |
|  | 2009/12 | Juvenile | Non-tuberculous | 6 | 0.33 | 0.096 | 0.8 | 9.7 | 0.438 |
|  |  |  | Tuberculous | 8 | 0.23 | 0.069 |  |  |  |
|  |  | Adults | Non-tuberculous | 4 | 0.12 | 0.049 | -0.5 | 7.3 | 0.646 |
|  |  |  | Tuberculous | 17 | 0.15 | 0.039 |  |  |  |
| **IGSF21** | 2002/06 | Juvenile | Non-tuberculous | 8 | 0.55 | 0.065 | -0.5 | 3.7 | 0.634 |
|  |  |  | Tuberculous | 4 | 0.66 | 0.193 |  |  |  |
|  |  | Adults | Non-tuberculous | 4 | 0.43 | 0.064 | -1.8 | 9.7 | 0.096 |
|  |  |  | Tuberculous | 10 | 0.60 | 0.069 |  |  |  |
|  | 2009/12 | Juvenile | Non-tuberculous | 6 | 0.36 | 0.107 | 0.8 | 11.3 | 0.459 |
|  |  |  | Tuberculous | 8 | 0.24 | 0.100 |  |  |  |
|  |  | Adults | Non-tuberculous | 4 | 0.09 | 0.051 | -0.5 | 6.6 | 0.637 |
|  |  |  | Tuberculous | 17 | 0.12 | 0.036 |  |  |  |
| **NTRK** | 2002/06 | Juvenile | Non-tuberculous | 7 | 0.02 | 0.006 | 1.5 | 7.5 | 0.176 |
|  |  |  | Tuberculous | 3 | 0.01 | 0.005 |  |  |  |
|  |  | Adults | Non-tuberculous | 4 | 0.01 | 0.006 | -2.1 | 6.7 | 0.074 |
|  |  |  | Tuberculous | 10 | 0.03 | 0.005 |  |  |  |
|  | 2009/12 | Juvenile | Non-tuberculous | 6 | 0.01 | 0.006 | 0.3 | 10.8 | 0.754 |
|  |  |  | Tuberculous | 8 | 0.01 | 0.005 |  |  |  |
|  |  | Adults | Non-tuberculous | 4 | 0.00 | 0.002 | -0.8 | 13.2 | 0.435 |
|  |  |  | Tuberculous | 15 | 0.01 | 0.003 |  |  |  |
| **MUT** | 2002/06 | Juvenile | Non-tuberculous | 8 | 0.94 | 0.078 | -0.6 | 10.0 | 0.541 |
|  |  |  | Tuberculous | 4 | 1.00 | 0.055 |  |  |  |
|  |  | Adults | Non-tuberculous | 4 | 1.09 | 0.118 | -1.3 | 9.9 | 0.233 |
|  |  |  | Tuberculous | 10 | 1.31 | 0.130 |  |  |  |
|  | 2009/12 | Juvenile | Non-tuberculous | 6 | 1.16 | 0.205 | 0.6 | 11.4 | 0.576 |
|  |  |  | Tuberculous | 8 | 1.00 | 0.194 |  |  |  |
|  |  | Adults | Non-tuberculous | 4 | 0.67 | 0.173 | -0.3 | 3.5 | 0.780 |
|  |  |  | Tuberculous | 17 | 0.72 | 0.051 |  |  |  |
| **C3** | 2002/06 | Juvenile | Non-tuberculous | 4 | 0.00 | 0.000 | 0.7 | 3.4 | 0.533 |
|  |  |  | Tuberculous | 3 | 0.00 | 0.000 |  |  |  |
|  |  | Adults | Non-tuberculous | 3 | 0.00 | 0.001 | -0.3 | 7.4 | 0.776 |
|  |  |  | Tuberculous | 7 | 0.00 | 0.001 |  |  |  |
|  | 2009/12 | Juvenile | Non-tuberculous | 6 | 0.01 | 0.006 | -0.4 | 9.6 | 0.716 |
|  |  |  | Tuberculous | 8 | 0.01 | 0.005 |  |  |  |
|  |  | Adults | Non-tuberculous | 4 | 0.02 | 0.010 | 0.3 | 3.5 | 0.789 |
|  |  |  | Tuberculous | 17 | 0.01 | 0.003 |  |  |  |

**Table S5**: Expression profile (normalized Ct values) of genes associated to SNPs with the highest allele frequency differences in the standard and/or stratified genome-wide analyses (GWAS). Differences between juvenile and adults animals were quantified for uninfected and infected individuals with *Mycobacterium tuberculosis complex* (MTC) and for 2002/06 and 2009/12 time-period using an independent t-test.

| **Gene** | **Tuberculosis** | **Time-period** | **Age class** | **N** | **Mean** | **SE** | **t** | **df** | ***p-value*** |
| --- | --- | --- | --- | --- | --- | --- | --- | --- | --- |
| **LOC102164072** | Non-tuberculous | 2002/06 | Juvenile | 7 | 0.56 | 0.052 | -0.9 | 4.6 | 0.434 |
|  |  |  | Adults | 4 | 0.66 | 0.102 |  |  |  |
|  |  | 2009/12 | Juvenile | 6 | 0.45 | 0.134 | 1.8 | 7.1 | 0.112 |
|  |  |  | Adults | 4 | 0.18 | 0.067 |  |  |  |
|  | Tuberculous | 2002/06 | Juvenile | 3 | 0.57 | 0.142 | -0.7 | 3.9 | 0.550 |
|  |  |  | Adults | 9 | 0.68 | 0.093 |  |  |  |
|  |  | 2009/12 | Juvenile | 8 | 0.37 | 0.106 | 1.2 | 11.8 | 0.258 |
|  |  |  | Adults | 17 | 0.22 | 0.061 |  |  |  |
| **RXFP1** | Non-tuberculous | 2002/06 | Juvenile | 8 | 0.79 | 0.078 | -0.2 | 3.7 | 0.838 |
|  |  |  | Adults | 4 | 0.85 | 0.239 |  |  |  |
|  |  | 2009/12 | Juvenile | 6 | 0.86 | 0.261 | 1.6 | 7.5 | 0.143 |
|  |  |  | Adults | 4 | 0.37 | 0.153 |  |  |  |
|  | Tuberculous | 2002/06 | Juvenile | 4 | 0.65 | 0.114 | -2.1 | 11.6 | 0.055 |
|  |  |  | Adults | 10 | 1.07 | 0.165 |  |  |  |
|  |  | 2009/12 | Juvenile | 8 | 0.56 | 0.148 | 1.0 | 13.2 | 0.325 |
|  |  |  | Adults | 17 | 0.38 | 0.097 |  |  |  |
| **ATP9A** | Non-tuberculous | 2002/06 | Juvenile | 0 |  |  |  |  |  |
|  |  |  | Adults | 1 | 0.00 |  |  |  |  |
|  |  | 2009/12 | Juvenile | 5 | 0.02 | 0.008 | -1.2 | 5.3 | 0.297 |
|  |  |  | Adults | 4 | 0.03 | 0.013 |  |  |  |
|  | Tuberculous | 2002/06 | Juvenile | 0 |  |  |  |  |  |
|  |  |  | Adults | 3 | 0.00 | 0.000 |  |  |  |
|  |  | 2009/12 | Juvenile | 8 | 0.03 | 0.009 | 0.3 | 9.5 | 0.763 |
|  |  |  | Adults | 17 | 0.02 | 0.004 |  |  |  |
| **NFATC** | Non-tuberculous | 2002/06 | Juvenile | 2 | 0.04 | 0.017 | -1.0 | 1.4 | 0.471 |
|  |  |  | Adults | 2 | 0.08 | 0.039 |  |  |  |
|  |  | 2009/12 | Juvenile | 6 | 0.06 | 0.031 | -0.6 | 6.8 | 0.560 |
|  |  |  | Adults | 4 | 0.08 | 0.014 |  |  |  |
|  | Tuberculous | 2002/06 | Juvenile | 0 |  |  |  |  |  |
|  |  |  | Adults | 3 | 0.11 | 0.036 |  |  |  |
|  |  | 2009/12 | Juvenile | 8 | 0.06 | 0.018 | -1.2 | 15.4 | 0.239 |
|  |  |  | Adults | 17 | 0.09 | 0.014 |  |  |  |
| **BDNF/NT3** | Non-tuberculous | 2002/06 | Juvenile | 8 | 0.70 | 0.097 | 0.1 | 6.4 | 0.916 |
|  |  |  | Adults | 4 | 0.68 | 0.129 |  |  |  |
|  |  | 2009/12 | Juvenile | 6 | 0.33 | 0.096 | 1.9 | 7.2 | 0.094 |
|  |  |  | Adults | 4 | 0.12 | 0.049 |  |  |  |
|  | Tuberculous | 2002/06 | Juvenile | 4 | 0.89 | 0.182 | -0.8 | 5.5 | 0.457 |
|  |  |  | Adults | 10 | 1.06 | 0.114 |  |  |  |
|  |  | 2009/12 | Juvenile | 8 | 0.23 | 0.069 | 1.0 | 11.6 | 0.319 |
|  |  |  | Adults | 17 | 0.15 | 0.039 |  |  |  |
| **IGSF21** | Non-tuberculous | 2002/06 | Juvenile | 8 | 0.55 | 0.065 | 1.4 | 8.5 | 0.206 |
|  |  |  | Adults | 4 | 0.43 | 0.064 |  |  |  |
|  |  | 2009/12 | Juvenile | 6 | 0.36 | 0.107 | 2.2 | 6.9 | 0.060 |
|  |  |  | Adults | 4 | 0.09 | 0.051 |  |  |  |
|  | Tuberculous | 2002/06 | Juvenile | 4 | 0.66 | 0.193 | 0.3 | 3.8 | 0.798 |
|  |  |  | Adults | 10 | 0.60 | 0.069 |  |  |  |
|  |  | 2009/12 | Juvenile | 8 | 0.24 | 0.100 | 1.2 | 8.9 | 0.276 |
|  |  |  | Adults | 17 | 0.12 | 0.036 |  |  |  |
| **NTRK** | Non-tuberculous | 2002/06 | Juvenile | 7 | 0.02 | 0.006 | 1.1 | 7.7 | 0.323 |
|  |  |  | Adults | 4 | 0.01 | 0.006 |  |  |  |
|  |  | 2009/12 | Juvenile | 6 | 0.01 | 0.006 | 1.3 | 6.5 | 0.240 |
|  |  |  | Adults | 4 | 0.00 | 0.002 |  |  |  |
|  | Tuberculous | 2002/06 | Juvenile | 3 | 0.01 | 0.005 | -2.9 | 6.9 | 0.025 |
|  |  |  | Adults | 10 | 0.03 | 0.005 |  |  |  |
|  |  | 2009/12 | Juvenile | 8 | 0.01 | 0.005 | 0.5 | 12.2 | 0.660 |
|  |  |  | Adults | 15 | 0.01 | 0.003 |  |  |  |
| **MUT** | Non-tuberculous | 2002/06 | Juvenile | 8 | 0.94 | 0.078 | -1.1 | 5.8 | 0.332 |
|  |  |  | Adults | 4 | 1.09 | 0.118 |  |  |  |
|  |  | 2009/12 | Juvenile | 6 | 1.16 | 0.205 | 1.9 | 7.9 | 0.101 |
|  |  |  | Adults | 4 | 0.67 | 0.173 |  |  |  |
|  | Tuberculous | 2002/06 | Juvenile | 4 | 1.00 | 0.055 | -2.2 | 11.4 | 0.048 |
|  |  |  | Adults | 10 | 1.31 | 0.130 |  |  |  |
|  |  | 2009/12 | Juvenile | 8 | 1.00 | 0.194 | 1.4 | 8.0 | 0.200 |
|  |  |  | Adults | 17 | 0.72 | 0.051 |  |  |  |
| **C3** | Non-tuberculous | 2002/06 | Juvenile | 4 | 0.00 | 0.000 | -0.9 | 2.0 | 0.461 |
|  |  |  | Adults | 3 | 0.00 | 0.001 |  |  |  |
|  |  | 2009/12 | Juvenile | 6 | 0.01 | 0.006 | -0.6 | 5.4 | 0.562 |
|  |  |  | Adults | 4 | 0.02 | 0.010 |  |  |  |
|  | Tuberculous | 2002/06 | Juvenile | 3 | 0.00 | 0.000 | -1.1 | 6.0 | 0.301 |
|  |  |  | Adults | 7 | 0.00 | 0.001 |  |  |  |
|  |  | 2009/12 | Juvenile | 8 | 0.01 | 0.005 | -0.3 | 13.1 | 0.799 |
|  |  |  | Adults | 17 | 0.01 | 0.003 |  |  |  |

**Table S6:** Statistical power and size sample calculations results. The calculation was treated as a function of high-risk allele frequency and considering disease prevalence of 45%, complete linkage disequilibrium (D’=1), 5% type I error rate and a relative risk associated to minor allele frequency (MAF) genotype of 0.01. Conditions surpassing ≥80% statistical power are highlighted in gray. The power calculations are presented for i) the classical case-control analysis, during which individuals infected with MTC were compared with those that were uninfected, and *ii)* a case-control analyses of individuals from the lowest and highest periods of TB prevalence (2002/06 *vs.* 2009/12).

|  | **GWAS TB outcome** | | |  | **GWAS Time-period** | | |
| --- | --- | --- | --- | --- | --- | --- | --- |
|  | High-risk allele frequency | | |  | High-risk allele frequency | | |
|  | 0.05 | 0.10 | 0.15 |  | 0.05 | 0.10 | 0.15 |
| Statistical power | 80.8% | 95.8% | 98.7% |  | 67.9% | 89% | 95.5% |
| Number of cases required to achieve 80% power | 27 | 16 | 12 |  | 48 | 27 | 21 |
| Number of cases used in the GWAS | 28 | 28 | 28 |  | 36 | 36 | 36 |
| Number of cases used in the validation analyses | 48 | 48 | 48 |  | 56 | 56 | 56 |


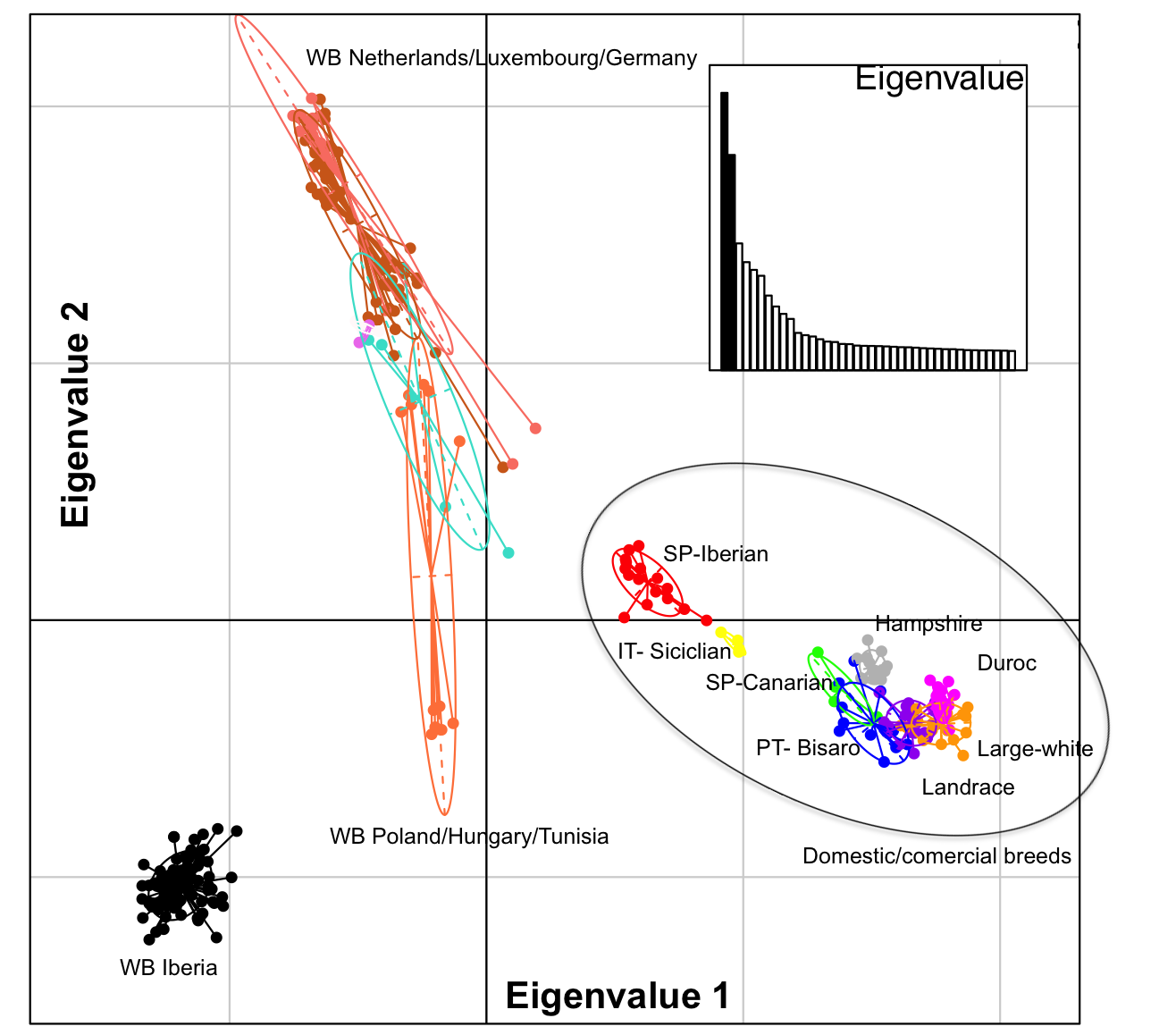


**Fig. S1** Plot showing the results of principal component analysis (PCA) performed with ADGENET software using 313 individuals genotyped for 15437 SNPs. This dataset was obtained for the population studied and the data available for commercial and Iberian breeds from literature.

**
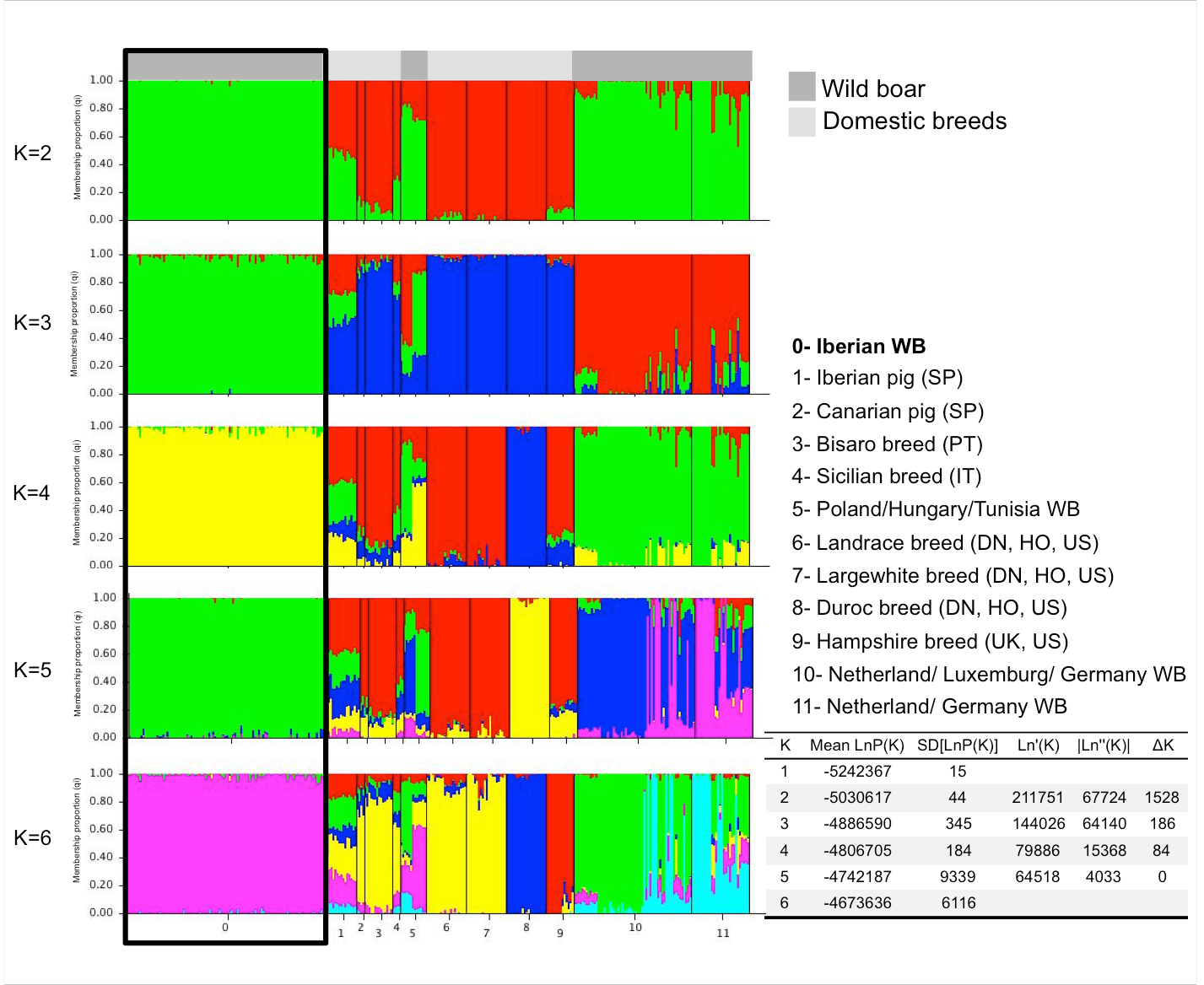
**

**Fig. S2** Plot showing the results of the Bayesian clusters analysis carried out with STRUCTURE software. Each line represents an individual and colors refers to the membership proportion assigned to each k cluster. Delta K (Δ*K*) results obtained in further Evanno analyses are also shown.


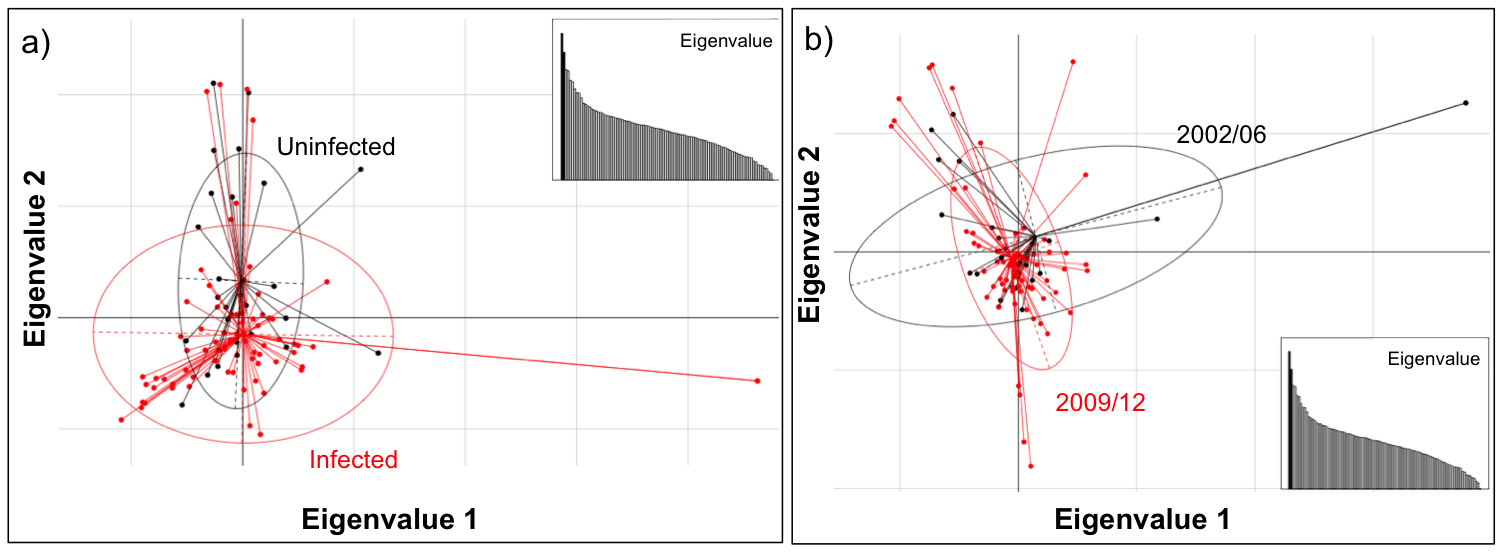


**Fig. S3** Plots showing the results of principal component analyses (PCA) performed with ADEGENET software using the sampled wild boar (n = 100) genotyped for 15437 SNPs. Individuals uninfected and infected with *Mycobacterium tuberculosis complex* (MTC) are shown on the left (a), while individuals from 2002/06 and 2009/12 are represented on the right (b).


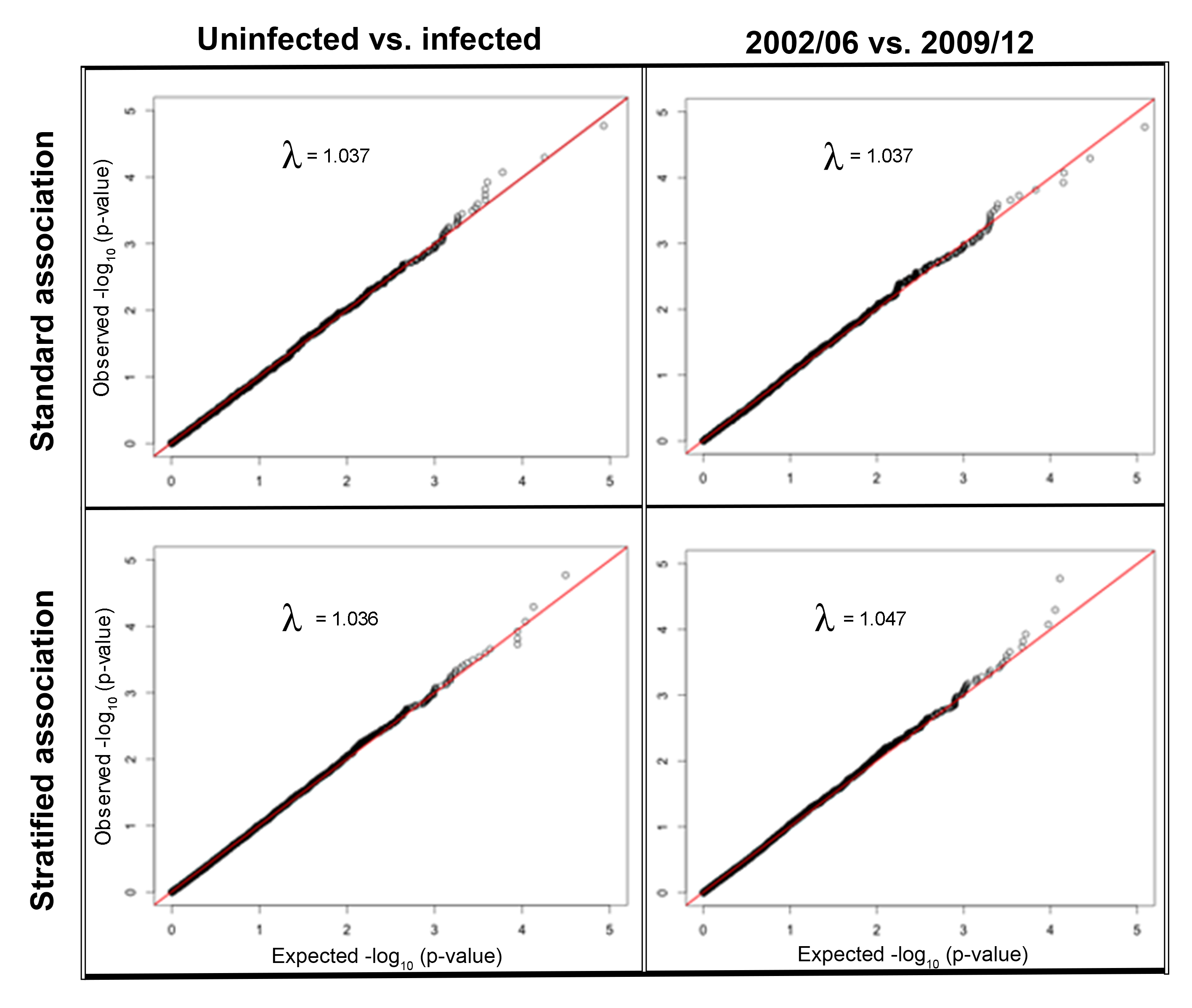


**Fig. S4** QQ-plots performed for genome-wide associations (GWAS). The genome inflation factor (λ) is depicted for each of the statistical models constructed.


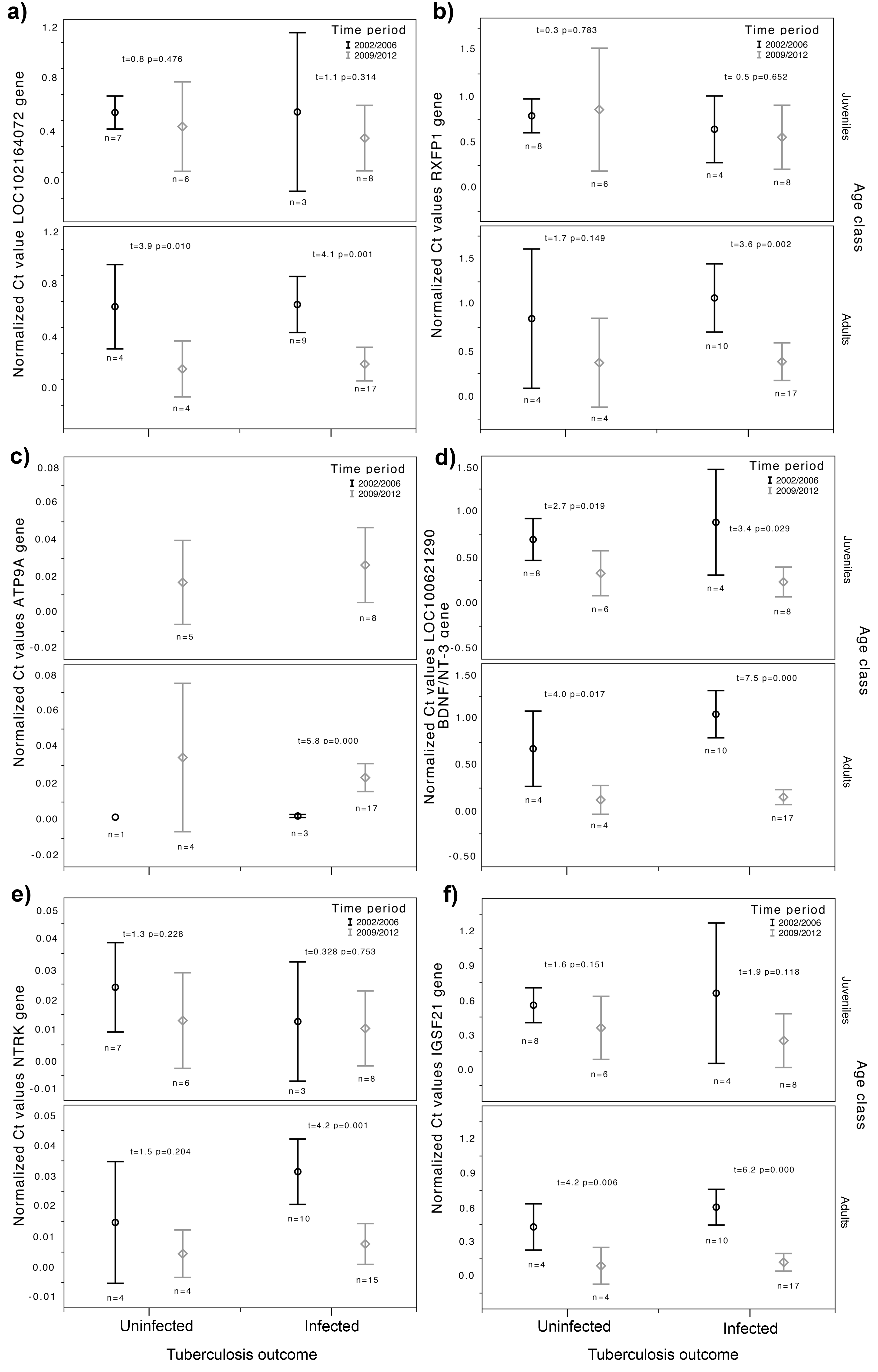


**Fig. S5** Plots showing the mRNA expression levels (average and confidence interval at 95%) by age class, TB outcome and time-period for the genes associated to SNPs with the highest allele frequency differences in the standard and/or stratified GWAS; a - *LOC102164072*, b - *RXFP1*, c - *ATP9A*, d - *LOC100621290/BDNF/NT-3*, e - *NTRK*, f - *IGSF21*).


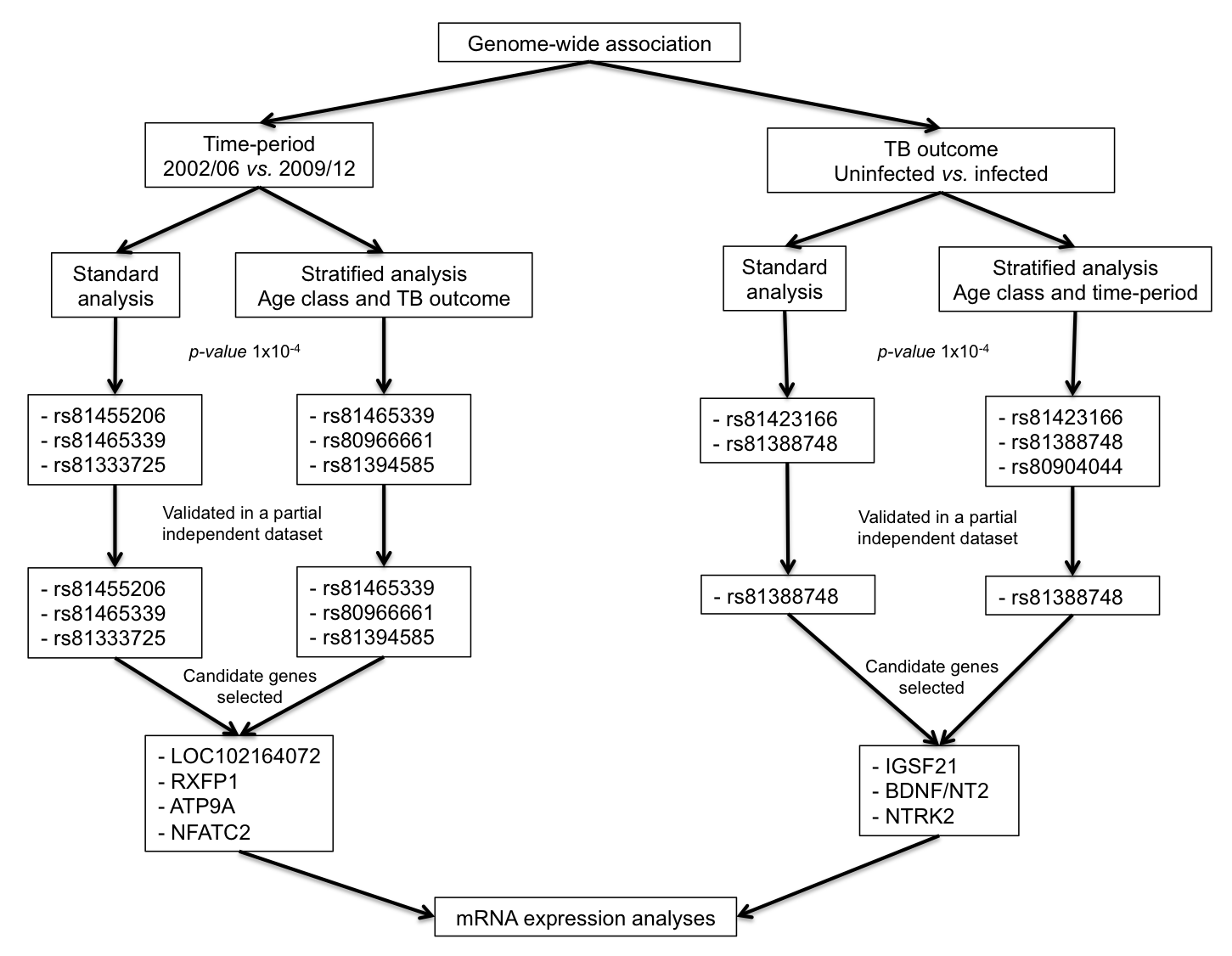


**Fig. S6** Workflow of genome-wide associations (GWAS) conducted in this study, and the candidate genes selected from the analyses.
